# Supplementary material for: ParSe 2.0: A web tool to identify drivers of protein phase separation at the proteome level
Source: Protein Sci. 2023 Sep 1;32(9):e4756. doi: 10.1002/pro.4756 (PMC10464302; doi:10.1002/pro.4756)
Supplement: Supplementary file 1 — Data S1: Supporting Information. [file PRO-32-e4756-s001.docx]

**Supporting Information for:**

**ParSe 2.0: A web tool to identify drivers of protein phase separation at the proteome level.**

*Colorado Wilson, Karen A. Lewis, Nicholas C. Fitzkee, Loren E. Hough, and Steven T. Whitten*

**Contents:**

Supporting Tables

S1. Proteins confirmed to exhibit homotypic phase separation behavior.

S2. Summary table for the set of proteins that exhibit homotypic phase separation behavior.

Supporting Figures

S1. Mutation effects on experimental *c_sat_* compared to the PS potential trained previously using experimental ∆*h°* from a mutant dataset.

Supporting References

**Supporting Tables**

**Table S1. Proteins confirmed to exhibit homotypic phase separation behavior.**

| **UniProtKB ID *^a^*** | **Gene name** | **Primary sequence** |
| --- | --- | --- |
| Q92804 | RBP56_HUMAN | MSDSGSYGQSGGEQQSYSTYGNPGSQGYGQASQSYSGYGQTTDSSYGQNYSGYSSYGQSQSGYSQSYGGYENQKQSSYSQQPYNNQGQQQNMESSGSQGGRAPSYDQPDYGQQDSYDQQSGYDQHQGSYDEQSNYDQQHDSYSQNQQSYHSQRENYSHHTQDDRRDVSRYGEDNRGYGGSQGGGRGRGGYDKDGRGPMTGSSGGDRGGFKNFGGHRDYGPRTDADSESDNSDNNTIFVQGLGEGVSTDQVGEFFKQIGIIKTNKKTGKPMINLYTDKDTGKPKGEATVSFDDPPSAKAAIDWFDGKEFHGNIIKVSFATRRPEFMRGGGSGGGRRGRGGYRGRGGFQGRGGDPKSGDWVCPNPSCGNMNFARRNSCNQCNEPRPEDSRPSGGDFRGRGYGGERGYRGRGGRGGDRGGYGGDRSGGGYGGDRSSGGGYSGDRSGGGYGGDRSGGGYGGDRGGGYGGDRGGGYGGDRGGGYGGDRGGYGGDRGGGYGGDRGGYGGDRGGYGGDRGGYGGDRGGYGGDRSRGGYGGDRGGGSGYGGDRSGGYGGDRSGGGYGGDRGGGYGGDRGGYGGKMGGRNDYRNDQRNRPY |
| P09651-2 | ROA1_HUMAN | MSKSESPKEPEQLRKLFIGGLSFETTDESLRSHFEQWGTLTDCVVMRDPNTKRSRGFGFVTYATVEEVDAAMNARPHKVDGRVVEPKRAVSREDSQRPGAHLTVKKIFVGGIKEDTEEHHLRDYFEQYGKIEVIEIMTDRGSGKKRGFAFVTFDDHDSVDKIVIQKYHTVNGHNCEVRKALSKQEMASASSSQRGRSGSGNFGGGRGGGFGGNDNFGRGGNFSGRGGFGGSRGGGGYGGSGDGYNGFGNDGSNFGGGGSYNDFGNYNNQSSNFGPMKGGNFGGRSSGPYGGGGQYFAKPRNQGGYGGSSSSSSYGSGRRF |
| D0PV95 | DDX3_CAEEL | MESNQSNNGGSGNAALNRGGRYVPPHLRGGDGGAAAAASAGGDDRRGGAGGGGYRRGGGNSGGGGGGGYDRGYNDNRDDRDNRGGSGGYGRDRNYEDRGYNGGGGGGGNRGYNNNRGGGGGGYNRQDRGDGGSSNFSRGGYNNRDEGSDNRGSGRSYNNDRRDNGGDGQNTRWNNLDAPPSRGTSKWENRGARDERIEQELFSGQLSGINFDKYEEIPVEATGDDVPQPISLFSDLSLHEWIEENIKTAGYDRPTPVQKYSIPALQGGRDLMSCAQTGSGKTAAFLVPLVNAILQDGPDAVHRSVTSSGGRKKQYPSALVLSPTRELSLQIFNESRKFAYRTPITSALLYGGRENYKDQIHKLRLGCHILIATPGRLIDVMDQGLIGMEGCRYLVLDEADRMLDMGFEPQIRQIVECNRMPSKEERITAMFSATFPKEIQLLAQDFLKENYVFLAVGRVGSTSENIMQKIVWVEEDEKRSYLMDLLDATGDSSLTLVFVETKRGASDLAYYLNRQNYEVVTIHGDLKQFEREKHLDLFRTGTAPILVATAVAARGLDIPNVKHVINYDLPSDVDEYVHRIGRTGRVGNVGLATSFFNDKNRNIARELMDLIVEANQELPDWLEGMSGDMRSGGGYRGRGGRGNGQRFGGRDHRYQGGSGNGGGGNGGGGGFGGGGQRSGGGGGFQSGGGGGRQQQQQQRAQPQQDWWS |
| H3BNZ4 | H3BNZ4_HUMAN | MASNDYTQQATQSYGAYPTQPGQGYSQQSSQPYGQQSYSGYSQSTDTSGYGQSSYSSYGQSQNTGYGTQSTPQGYGSTGGYGSSQSSQSSYGQQSSYPGYGQQPAPSSTSGSYGSSSQSSSYGQPQSGSYSQQPSYGGQQQSYGQQQSYNPPQGYGQQNQYNSSSGGGGGGGGGGNYGQDQSSMSSGGGSGGGYGNQDQSGGGGSGGYGQQDRGGRGRGGSGGGGGGGGGGYNRSSGGYEPRGRGGGRGGRGGMGPSGPRITS |
| O00571 | DDX3X_HUMAN | MSHVAVENALGLDQQFAGLDLNSSDNQSGGSTASKGRYIPPHLRNREATKGFYDKDSSGWSSSKDKDAYSSFGSRSDSRGKSSFFSDRGSGSRGRFDDRGRSDYDGIGSRGDRSGFGKFERGGNSRWCDKSDEDDWSKPLPPSERLEQELFSGGNTGINFEKYDDIPVEATGNNCPPHIESFSDVEMGEIIMGNIELTRYTRPTPVQKHAIPIIKEKRDLMACAQTGSGKTAAFLLPILSQIYSDGPGEALRAMKENGRYGRRKQYPISLVLAPTRELAVQIYEEARKFSYRSRVRPCVVYGGADIGQQIRDLERGCHLLVATPGRLVDMMERGKIGLDFCKYLVLDEADRMLDMGFEPQIRRIVEQDTMPPKGVRHTMMFSATFPKEIQMLARDFLDEYIFLAVGRVGSTSENITQKVVWVEESDKRSFLLDLLNATGKDSLTLVFVETKKGADSLEDFLYHEGYACTSIHGDRSQRDREEALHQFRSGKSPILVATAVAARGLDISNVKHVINFDLPSDIEEYVHRIGRTGRVGNLGLATSFFNERNINITKDLLDLLVEAKQEVPSWLENMAYEHHYKGSSRGRSKSSRFSGGFGARDYRQSSGASSSSFSSSRASSSRSGGGGHGSSRGFGGGGYGGFYNSDGYGGNYNSQGVDWWGN |
| Q9NQI0 | DDX4_HUMAN | MGDEDWEAEINPHMSSYVPIFEKDRYSGENGDNFNRTPASSSEMDDGPSRRDHFMKSGFASGRNFGNRDAGECNKRDNTSTMGGFGVGKSFGNRGFSNSRFEDGDSSGFWRESSNDCEDNPTRNRGFSKRGGYRDGNNSEASGPYRRGGRGSFRGCRGGFGLGSPNNDLDPDECMQRTGGLFGSRRPVLSGTGNGDTSQSRSGSGSERGGYKGLNEEVITGSGKNSWKSEAEGGESSDTQGPKVTYIPPPPPEDEDSIFAHYQTGINFDKYDTILVEVSGHDAPPAILTFEEANLCQTLNNNIAKAGYTKLTPVQKYSIPIILAGRDLMACAQTGSGKTAAFLLPILAHMMHDGITASRFKELQEPECIIVAPTRELVNQIYLEARKFSFGTCVRAVVIYGGTQLGHSIRQIVQGCNILCATPGRLMDIIGKEKIGLKQIKYLVLDEADRMLDMGFGPEMKKLISCPGMPSKEQRQTLMFSATFPEEIQRLAAEFLKSNYLFVAVGQVGGACRDVQQTVLQVGQFSKREKLVEILRNIGDERTMVFVETKKKADFIATFLCQEKISTTSIHGDREQREREQALGDFRFGKCPVLVATSVAARGLDIENVQHVINFDLPSTIDEYVHRIGRTGRCGNTGRAISFFDLESDNHLAQPLVKVLTDAQQDVPAWLEEIAFSTYIPGFSGSTRGNVFASVDTRKGKSTLNTAGFSSSQAPNPVDDESWD |
| Q15056 | IF4H_HUMAN | MADFDTYDDRAYSSFGGGRGSRGSAGGHGSRSQKELPTEPPYTAYVGNLPFNTVQGDIDAIFKDLSIRSVRLVRDKDTDKFKGFCYVEFDEVDSLKEALTYDGALLGDRSLRVDIAEGRKQDKGGFGFRKGGPDDRGMGSSRESRGGWDSRDDFNSGFRDDFLGGRGGSRPGDRRTGPPMGSRFRDGPPLRGSNMDFREPTEEERAQRPRLQLKPRTVATPLNQVANPNSAIFGGARPREEVVQKEQE |
| P14907 | NSP1_YEAST | MNFNTPQQNKTPFSFGTANNNSNTTNQNSSTGAGAFGTGQSTFGFNNSAPNNTNNANSSITPAFGSNNTGNTAFGNSNPTSNVFGSNNSTTNTFGSNSAGTSLFGSSSAQQTKSNGTAGGNTFGSSSLFNNSTNSNTTKPAFGGLNFGGGNNTTPSSTGNANTSNNLFGATANANKPAFSFGATTNDDKKTEPDKPAFSFNSSVGNKTDAQAPTTGFSFGSQLGGNKTVNEAAKPSLSFGSGSAGANPAGASQPEPTTNEPAKPALSFGTATSDNKTTNTTPSFSFGAKSDENKAGATSKPAFSFGAKPEEKKDDNSSKPAFSFGAKSNEDKQDGTAKPAFSFGAKPAEKNNNETSKPAFSFGAKSDEKKDGDASKPAFSFGAKPDENKASATSKPAFSFGAKPEEKKDDNSSKPAFSFGAKSNEDKQDGTAKPAFSFGAKPAEKNNNETSKPAFSFGAKSDEKKDGDASKPAFSFGAKSDEKKDSDSSKPAFSFGTKSNEKKDSGSSKPAFSFGAKPDEKKNDEVSKPAFSFGAKANEKKESDESKSAFSFGSKPTGKEEGDGAKAAISFGAKPEEQKSSDTSKPAFTFGAQKDNEKKTEESSTGKSTADVKSSDSLKLNSKPVELKPVSLDNKTLDDLVTKWTNQLTESASHFEQYTKKINSWDQVLVKGGEQISQLYSDAVMAEHSQNKIDQSLQYIERQQDELENFLDNFETKTEALLSDVVSTSSGAAANNNDQKRQQAYKTAQTLDENLNSLSSNLSSLIVEINNVSNTFNKTTNIDINNEDENIQLIKILNSHFDALRSLDDNSTSLEKQINSIKK |
| F8WC90 | F8WC90_HUMAN | ASTDYSTYSQAAAQQGYSAYTAQPTQGYAQTTQQAYGQQSYGTYGQPTDVSYTQAQTTATYGQTAYATSYGQPPTGYTTPTAPQAYSQPVQGYGTGAYDTTTATVTTTQASYAAQSAYGTQPAYPAYGQQPAATAPTRPQDGNKPTETSQPQSSTGGYNQPSLGYGQSNYSYPQVPGSYPMQPVTAPPSYPPTSYSSTQPTSYDQSSYSQQNTYGQPSSYGQQSSYGQQSSYGQQPPTSYPPQTGSYSQAPSQYSQQSSSYGQQSSFRQDHPSSMGVYGQESGGFSGPGEN |
| P31483 | TIA1_HUMAN | MEDEMPKTLYVGNLSRDVTEALILQLFSQIGPCKNCKMIMDTAGNDPYCFVEFHEHRHAAAALAAMNGRKIMGKEVKVNWATTPSSQKKDTSSSTVVSTQRSQDHFHVFVGDLSPEITTEDIKAAFAPFGRISDARVVKDMATGKSKGYGFVSFFNKWDAENAIQQMGGQWLGGRQIRTNWATRKPPAPKSTYESNTKQLSYDEVVNQSSPSNCTVYCGGVTSGLTEQLMRQTFSPFGQIMEIRVFPDKGYSFVRFNSHESAAHAIVSVNGTTIEGHVVKCYWGKETLDMINPVQQQNQIGYPQPYGQWGQWYGNAQQIGQYMPNGWQVPAYGMYGQAWNQQGFNQTQSSAPWMGPNYGVQPPQGQNGSMLPNQPSGYRVAGYETQ |
| P15502 | ELN_HUMAN | MAGLTAAAPRPGVLLLLLSILHPSRPGGVPGAIPGGVPGGVFYPGAGLGALGGGALGPGGKPLKPVPGGLAGAGLGAGLGAFPAVTFPGALVPGGVADAAAAYKAAKAGAGLGGVPGVGGLGVSAGAVVPQPGAGVKPGKVPGVGLPGVYPGGVLPGARFPGVGVLPGVPTGAGVKPKAPGVGGAFAGIPGVGPFGGPQPGVPLGYPIKAPKLPGGYGLPYTTGKLPYGYGPGGVAGAAGKAGYPTGTGVGPQAAAAAAAKAAAKFGAGAAGVLPGVGGAGVPGVPGAIPGIGGIAGVGTPAAAAAAAAAAKAAKYGAAAGLVPGGPGFGPGVVGVPGAGVPGVGVPGAGIPVVPGAGIPGAAVPGVVSPEAAAKAAAKAAKYGARPGVGVGGIPTYGVGAGGFPGFGVGVGGIPGVAGVPGVGGVPGVGGVPGVGISPEAQAAAAAKAAKYGAAGAGVLGGLVPGAPGAVPGVPGTGGVPGVGTPAAAAAKAAAKAAQFGLVPGVGVAPGVGVAPGVGVAPGVGLAPGVGVAPGVGVAPGVGVAPGIGPGGVAAAAKSAAKVAAKAQLRAAAGLGAGIPGLGVGVGVPGLGVGAGVPGLGVGAGVPGFGAGADEGVRRSLSPELREGDPSSSQHLPSTPSSPRVPGALAAAKAAKYGAAVPGVLGGLGALGGVGIPGGVVGAGPAAAAAAAKAAAKAAQFGLVGAAGLGGLGVGGLGVPGVGGLGGIPPAAAAKAAKYGAAGLGGVLGGAGQFPLGGVAARPGFGLSPIFPGGACLGKACGRKRK |
| P22626-2 | ROA2_HUMAN | MEREKEQFRKLFIGGLSFETTEESLRNYYEQWGKLTDCVVMRDPASKRSRGFGFVTFSSMAEVDAAMAARPHSIDGRVVEPKRAVAREESGKPGAHVTVKKLFVGGIKEDTEEHHLRDYFEEYGKIDTIEIITDRQSGKKRGFGFVTFDDHDPVDKIVLQKYHTINGHNAEVRKALSRQEMQEVQSSRSGRGGNFGFGDSRGGGGNFGPGPGSNFRGGSDGYGSGRGFGDGYNGYGGGPGGGNFGGSPGYGGGRGGYGGGGPGYGNQGGGYGGGYDNYGGGNYGSGNYNDFGNYNQQPSNYGPMKSGNFGGSRNMGGPYGGGNYGPGGSGGSGGYGGRSRY |
| P22232 | FBRL_XENLA | MRPGFSPRGGRGGFGDRGGFGGRGGFGDRGGFRGGSRGGFGGRGRGGDRGGRGGFRGGFSSPGRGGPRGGGRGGFGGGRGGFGAGRKVIVEPHRHEGIFICRGKEDALVTKNLVPGESVYGEKRISVEDGEVKTEYRAWNPFRSKIAAAILGGVDQIHIKPGVKVLYLGAASGTTVSHVSDVVGPEGLVYAVEFSHRSGRDLINVAKKRTNIIPVIEDARHPHKYRILVGMVDVVFADVAQPDQTRIVALNAHNFLKNGGHFVISIKANCIDSTAAPEAVFAAEVKKMQQENMKPQEQLTLEPYERDHAVVVGIYRPPPKQKK |
| G5EBV6 | PGL3_CAEEL | MEANKRQIVEVDGIKSYFFPHLAHYLASNDELLVNNIAQANKLAAFVLGATDKRPSNEEIAEMILPNDSSAYVLAAGMDVCLILGDDFRPKFDSGAEKLSQLGQAHDLAPIIDDEKKISMLARKTKLKKSNDAKILQVLLKVLGAEEAEEKFVELSELSSALDLDFDVYVLAKLLGFASEELQEEIEIIRDNVTDAFEACKPLLKKLMIEGPKIDSVDPFTQLLLTPQEESIEKAVSHIVARFEEASAVEDDESLVLKSQLGYQLIFLVVRSLADGKRDASRTIQSLMPSSVRAEVFPGLQRSVFKSAVFLASHIIQVFLGSMKSFEDWAFVGLAEDLESTWRRRAIAELLKKFRISVLEQCFSQPIPLLPQSELNNETVIENVNNALQFALWITEFYGSESEKKSLNQLQFLSPKSKNLLVDSFKKFAQGLDSKDHVNRIIESLEKSSSSEPSATAKQTTTSNGPTTVSTAAQVVTVEKMPFSRQTIPCEGTDLANVLNSAKIIGESVTVAAHDVIPEKLNAEKNDNTPSTASPVQFSSDGWDSPTKSVALPPKISTLEEEQEEDTTITKVSPQPQERTGTAWGSGDATPVPLATPVNEYKVSGFGAAPVASGFGQFASSNGTSGRGSYGGGRGGDRGGRGAYGGDRGRGGSGDGSRGYRGGDRGGRGSYGEGSRGYQGGRAGFFGGSRGGS |
| Q14011 | CIRBP_HUMAN | MASDEGKLFVGGLSFDTNEQSLEQVFSKYGQISEVVVVKDRETQRSRGFGFVTFENIDDAKDAMMAMNGKSVDGRQIRVDQAGKSSDNRSRGYRGGSAGGRGFFRGGRGRGRGFSRGGGDRGYGGNRFESRSGGYGGSRDYYSSRSQSGGYSDRSSGGSYRDSYDSYATHNE |
| D3KYQ3 | D3KYQ3_TETTH | MFGNTGGGGLFGNTQTQQTGGGLFGQPQQTQFGQTGATGGGLFGGATNTFGGGGGGGLFGGNNNQQTNPTAGGGIFGQGTTGLGGAPAQTGGGLFGAPQNNQQGGGLFGGGTTTGGGMFGNQANTQTGGGGLFGGPSQPTTQPPAFSLNNPTTGGGGLFGQPANTMGGNNGGLFGGQTNSFGANNNMLGNNNRPQGAGIFGGATTTAPTGNTGMFGGIGANNGGGGLFGMNNTNTNPTGGFGATNPTAGGGGLFGGGATTTGGGGLFGGGNTQGGGLLGTANTTAGGLLGGGFNMNNNTGGILGQTNNQFGLGSFGTNNNAAAAPFQPKASANGVLTKPNEKNLCYAISNGTDFCIFELALTQRKLVKAGQLKPGAQQAGGMFGQPAQGGNGLFGGGGAATTTPFGGAQNGNLFGGQNTQAQGGGLFGAPVNNAATGAGGGLFGAKPAATTTGGGLFGQMPAQTGGFLGNTATQPAGGGLFGGATTTQAPGGGGGGGLFGGNTTAATTGGGLFGGNTQTGGATGGLFGGQQPNNQGGLFLNTGNANNANTGGGLFGGATTTPATGGGLFGGSTNTQPGLATGGGLFGNNQGASQPAAQGGLFGGAAPQQNSLFGGATAGGQTGGLFGGATGATQQQGGGLFGQTASNPTQGGGLFGAANPGLGGAAAGTQVAQPGLNGFQSLIQLLEQTQNQNNDKFALDDDTEQNWVYGKLRDGYQGGSLGYFQGGDSYSQSGNKFIQLEQQSLYGYQKKKQAYNQFKNLAHRDEKEKKDEMKKLLSSFSSSFVGIGSEQKKQVNLSSQSQALPTRLQQRKLSGSSEISHVSEKLKQIEKSDLCIDFIKSRTSNRIRIPVKPRYTFGDVKQKILNELINDNEINVRNFRLQARLKGPNKAPKPLRDTDPVFQNKNLPFENIDYIELVERSGNISSLNATLNNNNNSFVLNNIPNDEIDDRNSLQNNSRPIDQKPLLKARSQRINLPFRFSSMPSVDEMEKFTEQQLSQVENFVLENEYARIVFQEPVNLYNLDLSQLIFEESRISLEQVPESDRLNKKCIIEFKKFGLKQLNSGKSKEEVKTQVERLVKKKNLQLVKPYDVDNTKLVYIQDKFY |
| Q387F2 | Q387F2_TRYB2 | MSAGFGGGFGQPAATGFGQQPTGGFGQAPQGGAFGQVAPAATGFGQPSQSAVTGGFGQTNTGGFGQPAATGFGQPAQGAVTGGFGQTNTGGFGQPAATGFGQPAQSAVTGGFGQTNTGGFGQPAQGGFGQTAAAANAFGQAGPSGGFGQTNTGGFGQQSNSGFGQAGRGATAGFGQPGTGFGQPATGGFGQATSASPFGQAAAGRGVGGGFGTAAGTVGGFGQPAATGGFGQTATTGGFGQPAQGAAAGGFGQPATGGFGQATSASPFGQAAAGRGVGGGFGTAAGTVGGFGQPAAPGGFGQTATAGGFGQPARGAAAGGFGQPATGGFGQATSASPFGQAAAGRGVGGGFGTAAGTVGGFGQPAATGGFGQTATTGGFGQPAQGANTFGQGTPSAGGFGQAGRGVTGGFGQTGVTGGFGQTATTGGFGQPAQGAATGGFGQAGRGAADGFGRPAQGAAAGGFGQPATGGFGQATSASPFGQAAAGRGVGGGFGTAAGTVGGFGQPAAPGGFGQTATTGGFGQPGRGAAAGGFGQPATGGLGLAGGSGFGAAAGAGGFGQQSTASPWGGGATPAAPVDVSLLNLPEFADKPYGNVLLFAPEKPPKKPISVGHTPSPAAPFNPISVPRYQQRIPVGIPAPTVSANVKLQPTPFSTSALSSVELRELLNASKVNLGVSDDVQKIDSPKVSRVVAAGEKPPSHDAAVFAPVCSKEEYILDPPLATLQGLAVRQLQEVYGFSVYRRDGKCSVHFLEPVNLVRCDIAEIVELRPSGEVKLYPCVQNPPSIGQGLNVKARVTVNGVIGTTSNDLLIRCQKEGNRFESYDVNTGTWVYVMNVGDDGQNEYDDADREVDIVETDTQVQDGEQFPVANDIADGGVRAISQLDTCAQLKRSDPPSPPFLQQQQQVPVLHQNEAVSALSFSRYAPFDDQSLILPQRTQRVSVLRQAVHRPAADTTSGISDFELPYTLPSPKKTPLCERKGPLVVKEPYAKAHGPVYVVKREDSKMYEMNASVVSRGAMASLSRSFRCGWCTGGRLVAPMFAWVRDGTESRHSVDEVLGSRVVMSTVYFAHATSKHYLQSCAISVLRALCRHAHRVDASSDKEGFFPLLELGLCRDTGSTSLSTEKLREVIAAVDAVRFDGASAVGESTARQAKTILSLLDALYGLPDADEADKNAITERRYLTQLRRRNLNSWLKTELEYMDLWTDLDVDANPSQKLLRKLLCGKLREASSVAKALGSTELSRVVGICGEGNHFGSYVQTSDNSCIDEALGIRERVVSLLSGIVEPFVSQPQYAWAEDEKGGRTVAKVPLAATWKQLLGVFAFYGCTPDSSAEETIDCFLKRLRAPTSRRENSFPPYAERISADKLGTGRGRSFVSLGEWFPDAALSLLEGFAMGVAPAATALHPHSSSYCATDYLTPFIIIVSVRALKLQRTDTYDDAETKALLGFAAALECLSDAWFWALLPLHMIVDDKCRAVAVEQCLRRNAHRFQGGACRTNTDYVHLAELLKINARLLDVEMLLEKVPADAPVNAPSIRTHSSLQEALHRFSRGFTKR |
| Q64K55 | Q64K55_ARGTR | SSALFNAGVLNASNIDTLGSRVLSALLNGVSSAAQGLGINVDSGSVQSDISSSSSFLSTSSSSASYSQASASSTSGAGYTGPSGPSTGPSGYPGPLGGGAPFGQSGFGGSDGPQGGFGATGGASAGLISRVANALANTSTLRTVLRTGVSQQIASSVVQRAAQSLASTLGVDGNNLARFAVQAVSRLPAGSDTSAYAQAFSSALFNAGVLNASNIDTLGSRVLSALLNGVSSAAQGLGINVDSGSVQSDISSSSSFLSTSSSSASYSQASASSTSGAGYTGPSGPSTGPSGYPGLLGGGAPFGQSGFGGSDGPQGGFGATGGASAGLISRVANALANTSTLRTVLRTGVSQQIASSVVQRAAQSLASTLGVDGNNLARFAVQAVSRLPAGSDTSAYAQAFSSALFNAGVLNASNIDTLGSRVLSALLNGVSSAAQGLGINVDSGSVQSDISSSSSFLSTSSSSASYSQASASSTSGAGYTGPSGPSTGPSGYPGPLGGGAPFGQSGFGGSDGPQGGFGATGGASAGLISRVANALANTSTLRTVLRTGVSQQIASSVVQRAAQSLASTLGVDGNNLARFAVQAVSRLPAGSDTSAYAQAFSSALFNAGVLNASNIDTLGSRVLSALLNGVSSAAQGLGINVDSGSVQSDISSSSSFLSTSSSSASYSQASASSTSGAGYTGPSGPSTGPSGYPGPLGGGAPFGQSGFGGSAGPQGGFGATGGASAGLISRVANALANTSTLRTVLRTGVSQQIASSVVQRAAQSLASTLGVDGNNLARFAVQAVSRLPAGSDTSAYAQAFSSALFNAGVLNASNIDTLGSRVLSALLNGVSSAAQGLGINVDSGSVQSDISSSSSFLSTSSSSASYSQASASSTSGAGYTGPSGPSTGPSGYPGPLGGGAPFGQSGFGGSAGPQGGFGATGGASAGLISRVANALANTSTLRTVLRTGVSQQIASSVVQRAAQSLASTLGVDGNNLARFAVQAVSRLPAGSDTSAYAQAFSSALFNAGVLNASNIDTLGSRVLSALLNGVSSAAQGLGINVDSGSVQSDISSSSSFLSTSSSSASYSQASASSTSGTGYTGPSGPSTGPSGYPGPLGGGAPFGQSGFGGSAGPQGGFGATGGASAGLISRVANALANTSTLRTVLRTGVSQQIASSVVQRAAQSLASTLGVDGNNLARFAVQAVSRLPAGSDTSAYAQAFSSALFNAGVLNASNIDTLGSRVLSALLNGVSSAAQGLGINVDSGSVQSDISSSSSFLSTSSSSASYSQASASSTSGAGYTGPSGPSTGPSGYPGPLGGGAPFGQSGFGGSAGPQGGFGATGGASAGLISRVANALANTSTLRTVLRTGVSQQIASSVVQRAAQSLASTLGVDGNNLARFAVQAVSRLPAGSDTSAYAQAFSSALFNAGVLNASNIDTLGSRVLSALLNGVSSAAQGLGINVDSGSVQSDISSSSSFLSTSSSSASYSQASASSTSGAGYTGPSGPSTGPSGYPGPLGGGAPFGQSGFGGSDGPQGGFGATGGASAGLISRVANALANTSTLRTVLRTGVSQQIASSVVQRAAQSLASTLGVDGNNLARFAVQAVSRLPAGSDTSAYAQAFSSALFNAGVLNASNIDTLGSRVLSALLNGVSSAAQGLGINVDSGSVQSDISSSSSFLSTSSSSASYSQASASSTSGAGYTGPSGPSTGPSGYPGPLGGGAPFGQSGFGGSAGPQGGFGATGGASAGLISRVANALANTSTLRTVLRTGVSQQIASSVVQRAAQSLASTLGVDGNNLARFAVQAVSRLPAGSDTSAYAQAFSSALFNAGVLNASNIDTLGSRVLSALLNGVSSAAQGLGINVDSGSVQSDISSSSSFLSTSSSSASYSQASASSTSGAGYTGPSGPSTGPSGYPGPLGGGAPFGQSGFGGSAGPQGGFGATGGASAGLISRVANALANTSTLRTVLRTGVSQQIASSVVQRAAQSLASTLGVDGNNLARFAVQAVSRLPAGSDTSAYAQAFSSALFNAGVLNASNIDTLGSRVLSALLNGVSSAAQGLGINVDSGSVQSDISSSSSFLSTSSSSASYSQASASSTSGAGYTGPSGPSTGPSGYPGPLGGGAPFGQSGFGGSAGPQGGFGATGGASAGLISRVANALANTSTLRTVLRTGVSQQIASSVVQRAAQSLASTLGVDGNNLARFAVQAVSRLPAGSDTSAYAQAFSSALFNAGVLNASNIDTLGSRVLSALLNGVSSAAQGLGINVDSGSVQSDISSSSSFLSTSSSSASYSQASASSTSGAGYTGPSGPSTGPSGYPGPLGGGAPFGQSGFGGSAGPQGGFGATGGASAGLISRVANALANTSTLRTVLRTGVSQQIASSVVQRAAQSLASTLGVDGNNLARFAVQAVSRLPAGSDTSAYAQAFSSALFNAGVLNASNIDTLGSRVLSALLNGVSSAAQGLGINVDSGSVQSDISSSSSFLSTSSSSASYSQALASSTSGAGYTGPSGPSTGPSGYPGPLGGGAPFGQSGFGGSAGPQGGFGATGGASAGLISRVANALANTSTLRTVLRTGVSQQIASSVVQRAAQSLASTLGVDGNNLARFAVQAVSRLPAGSDTSAYAQAFSSALFNAGVLNASNIDTLGSRVLSALLNGVSSAAQGLGINVDSGSVQSDISSSSSFLSTSSSSASYSQASASSTSGAGYTGPSGPSTGPSGYPGPLSGGASFGSGQSSFGQTSAFSASGAGQSAGVSVISSLNSPVGLRSASAASRLSQLTSSITNAVGANGVDANSLARSLQSSFSALRSSGMSSSDAKIEVLLETIVGLLQLLSNTQVRGVNPATASSVANSAARSFELVLA |
| Q02629 | NU100_YEAST | MFGNNRPMFGGSNLSFGSNTSSFGGQQSQQPNSLFGNSNNNNNSTSNNAQSGFGGFTSAAGSNSNSLFGNNNTQNNGAFGQSMGATQNSPFGSLNSSNASNGNTFGGSSSMGSFGGNTNNAFNNNSNSTNSPFGFNKPNTGGTLFGSQNNNSAGTSSLFGGQSTSTTGTFGNTGSSFGTGLNGNGSNIFGAGNNSQSNTTGSLFGNQQSSAFGTNNQQGSLFGQQSQNTNNAFGNQNQLGGSSFGSKPVGSGSLFGQSNNTLGNTTNNRNGLFGQMNSSNQGSSNSGLFGQNSMNSSTQGVFGQNNNQMQINGNNNNSLFGKANTFSNSASGGLFGQNNQQQGSGLFGQNSQTSGSSGLFGQNNQKQPNTFTQSNTGIGLFGQNNNQQQQSTGLFGAKPAGTTGSLFGGNSSTQPNSLFGTTNVPTSNTQSQQGNSLFGATKLTNMPFGGNPTANQSGSGNSLFGTKPASTTGSLFGNNTASTTVPSTNGLFGNNANNSTSTTNTGLFGAKPDSQSKPALGGGLFGNSNSNSSTIGQNKPVFGGTTQNTGLFGATGTNSSAVGSTGKLFGQNNNTLNVGTQNVPPVNNTTQNALLGTTAVPSLQQAPVTNEQLFSKISIPNSITNPVKATTSKVNADMKRNSSLTSAYRLAPKPLFAPSSNGDAKFQKWGKTLERSDRGSSTSNSITDPESSYLNSNDLLFDPDRRYLKHLVIKNNKNLNVINHNDDEASKVKLVTFTTESASKDDQASSSIAASKLTEKAHSPQTDLKDDHDESTPDPQSKSPNGSTSIPMIENEKISSKVPGLLSNDVTFFKNNYYISPSIETLGNKSLIELRKINNLVIGHRNYGKVEFLEPVDLLNTPLDTLCGDLVTFGPKSCSIYENCSIKPEKGEGINVRCRVTLYSCFPIDKETRKPIKNITHPLLKRSIAKLKENPVYKFESYDPVTGTYSYTIDHPVLT |
| Q02630 | NU116_YEAST | MFGVSRGAFPSATTQPFGSTGSTFGGQQQQQQPVANTSAFGLSQQTNTTQAPAFGNFGNQTSNSPFGMSGSTTANGTPFGQSQLTNNNASGSIFGGMGNNTALSAGSASVVPNSTAGTSIKPFTTFEEKDPTTGVINVFQSITCMPEYRNFSFEELRFQDYQAGRKFGTSQNGTGTTFNNPQGTTNTGFGIMGNNNSTTSATTGGLFGQKPATGMFGTGTGSGGGFGSGATNSTGLFGSSTNLSGNSAFGANKPATSGGLFGNTTNNPTNGTNNTGLFGQQNSNTNGGLFGQQQNSFGANNVSNGGAFGQVNRGAFPQQQTQQGSGGIFGQSNANANGGAFGQQQGTGALFGAKPASGGLFGQSAGSKAFGMNTNPTGTTGGLFGQTNQQQSGGGLFGQQQNSNAGGLFGQNNQSQNQSGLFGQQNSSNAFGQPQQQGGLFGSKPAGGLFGQQQGASTFASGNAQNNSIFGQNNQQQQSTGGLFGQQNNQSQSQPGGLFGQTNQNNNQPFGQNGLQQPQQNNSLFGAKPTGFGNTSLFSNSTTNQSNGISGNNLQQQSGGLFQNKQQPASGGLFGSKPSNTVGGGLFGNNQVANQNNPASTSGGLFGSKPATGSLFGGTNSTAPNASSGGIFGSNNASNTAATTNSTGLFGNKPVGAGASTSAGGLFGNNNNSSLNNSNGSTGLFGSNNTSQSTNAGGLFQNNTSTNTSGGGLFSQPSQSMAQSQNALQQQQQQQRLQIQNNNPYGTNELFSKATVTNTVSYPIQPSATKIKADERKKASLTNAYKMIPKTLFTAKLKTNNSVMDKAQIKVDPKLSISIDKKNNQIAISNQQEENLDESILKASELLFNPDKRSFKNLINNRKMLIASEEKNNGSQNNDMNFKSKSEEQETILGKPKMDEKETANGGERMVLSSKNDGEDSATKHHSRNMDEENKENVADLQKQEYSEDDKKAVFADVAEKDASFINENYYISPSLDTLSSYSLLQLRKVPHLVVGHKSYGKIEFLEPVDLAGIPLTSLGGVIITFEPKTCIIYANLPNRPKRGEGINVRARITCFNCYPVDKSTRKPIKDPNHQLVKRHIERLKKNPNSKFESYDADSGTYVFIVNHAAEQT |
| F4ID16 | NU98B_ARATH | MFGSSNNNPFGQSSISSPFGTQTHSLFGQTNNNASNNPFATKPFGTSTPFGAQTGSSMFGGTSTGVFGAPQTSSPFGASPQAFGSSTQAFGASSTPSFGSSNSPFGGTSTFGQKSFGLSTPQSSPFGSTTQQSQPAFGNSTFGSSTPFGASTTPAFGASSTPAFGVSNTSGFGATNTPGFGATNTTGFGGSSTPGFGASSTPAFGSTNTPAFGASSTPLFGSSSSPAFGASPAPAFGSSGNAFGNNTFSSGGAFGSSSTPTFGASNTSAFGASSSPSFNFGSSPAFGQSTSAFGSSSFGSTQSSLGSTPSPFGAQGAQASTSTFGGQSTIGGQQGGSRVIPYAPTTDTASGTESKSERLQSISAMPAHKGKNMEELRWEDYQRGDKGGQRSTGQSPEGAGFGVTNSQPSIFSTSPAFSQTPVNPTNPFSQTTPTSNTNFSPSFSQPTTPSFGQPTTPSFRSTVSNTTSVFGSSSSLTTNTSQPLGSSIFGSTPAHGSTPGFSIGGFNNSQSSPLFGSNPSFAQNTTPAFSQTSPLFGQNTTPALGQSSSVFGQNTNPALVQSNTFSTPSTGFGNTFSSSSSLTTSISPFGQITPAVTPFQSAQPTQPLGAFGFNNFGQTQIANTTDIAGAMGTFSQGNFKQQPALGNSAVMQPTPVTNPFGTLPALPQISIAQGGNSPSIQYGISSMPVVDKPAPVRVSPLLTSRHLLQRRVRLPTRKYRPSDDGPKVPFFSDEEENSSTPKADAFFIPRENPRALFIRPVERVKSEHPKDSPTPLQENGKRSNGVTNGANHETKDNGAIREAPPVKVNQKQNGTHENHGGDKNGSHSSPSGADIESLMPKLHHSEYFTEPRIQELAAKERVEQGYCKRVKDFVVGRHGYGSIKFLGETDVCRLDLEMVVQFKNREVNVYMDESKKPPVGQGLNKPAVVTLLNIKCMDKKTGTQVMEGERLDKYKEMLKRKAGEQGAQFVSYDPVNGEWTFKVEHFSSYKLGDEYDV |
| Q54EQ8 | NUP98_DICDI | MFGGQFGSFGAKPAATASPFGAPSAAPTTSLFGSTAPSSGFGGFGSTAQTTQPTTGGFGGFGGFGGATTTQQPAASPFGGGGTGGSGLFGSSAQTTTQQPGASPFGGGFGTTTTTTTQQPGASPFGGTGGGLFGSSAQTTTQQQGASPFGGFGGATTTQPSLLSGATGGFGGFGGSTTSGTQLGGGGGATSGAFGGSSSPFGGSGGATTSSPFGGGGGSFGATTQKQYGTPIPYQQTTIEGNTFVSISAMPQYNDRSFEELRFEDITHRKDIVYKTGGGSGGGNSLFGSTPTTQPSSPFGAQTTTQTTGGLFGGQTTTSPFGGQTSATPGSSLFGSTQPTQQQTSGGLFGSVQPTQQQAGGGLFGSMPSTGGSSLFGSTQPTQQQTGGAQPTQSLFGGQTQTTTSPFGSQTSTPFGQPQQTNTGSGLFGAQQTQQTNTGGGLFGAQPTQQTSGGGLFGTQPTSGTGLFGTSPTAGGTGLFGTTQPTSQGTGLFGTTQPTTQGTGLFGTSPTSGTGLFGSTPTSGTGLFGSTPTSGTGLFGSAQPPQNQQSQTSLFGNTGTGATNTGTGLFGSAQPSSNPGGGLFGSAQPSTTTGGLFGSNQPTAQPTTSLFGNTTGSVGGLGATPNITSGLFGSNPAQTGGLFGSTQPTTQTSLFGNTGSTGGLGAQNGGGLFGNLSQPTATAGQGLSGGLFGNLSQPTATAGQGLSSGGLFGNTLLGQPSTQGLSSALPTLGLGLMGGQPQQTQQLPQGSLMLQQTQQPLQQQPLQQQQQPLQQSTIQLNNQINSASPYFPISSPAPFATFVKDLTSTSKVVSPPSYTQRSLSHHGYIPKSTTKLVPRRGPNNVDLGFSVIQNQNGLFPIDKFITKHSKSLNINTTNETEDTLRSLNTKSSSLFNNNNNNNNNNNNRNVNTNVNDYQNNGLPSSSLYNSNINQLSNNNNNNNNIYNNNNNNNINNNNNNNNISTQFNLRNNQSSSDNLNNDKSLSSSSSNKSQQQQQKEQKEEQPPKPIKEKEFINPNAPKLTRDGYQCVPSIKELSKKTDKELSSVQGFTISRDGCGSIYFPGSTNLVALDLDDIVDIEPREVSVYKDEETKPEIGYGLNRDAVVTLENCWPKNKNGEVVKEDGTILDKYENALKKVSAKSDCGFVSYSRSNGTWVFTVKHFSKYSAPDFDEDDQQMQQQTQQKQQQTQPSKVTFQQPSTKLTKPKFTANLDNFDSDSETSSGDENQDEMVPQKKTPFIKRVSNRESGLFDTPSVVPMSEKIETTPSKIARVSEPTSQSSRMSNNALKFSTFNPQQQQLQSSRFKSTGLSILSNPVKNLIQSDVNNEQSMFSNTTTTSTTRIQPLPSQQHLVQPIPTTISLNKNYFSKVRIDPQVYDRIVPKEESITNQYRMKNERLNHSTQDVSLFMRRSFRVGWAPGGKLISITKSSFKNLLIKKLPTDTKEDKKESIIKFLKNHHSHSSLVPENLKSIGWFSISNVQEQIESQLTLNVPSSQSVYYNRIWSLISNLWGNVLKGNGSKYINTNYSEDTIRKLNLNQWLKDVIAPLLRDEMDSLRKKTNSNYLEQIFSYLSAKQIKEASDLANENKDFRLATMMSQIWSSSESGKELILKQLTTYHSNGSDEFINEKRLEILHLIAGSVNKIYKNLNDWIRCFAVSFWFKYSLEYSIEDSVENFERSFNAHRSVYPLPPYLIKSTSTNSKQIEEQQHYYDICFLLLKLFAVNRGSSHFDKFKNIFYPENIGQDLLDYHLSWNLYTVLKSIPSLNKQPDLVNASNLHSSFALQLERLGLWQWSIYVLLHTPDQSNHVREEAVKSLIARAAPVITSEDRVFLTTKLHIPEIWIDEAKAWYSGYDCNNDIYDQIDALFKSYQYTKIHDIIFSNIGPNYIIQKRYHSLKDLLIRLEPHSSFISTWRYGGSIFLEFADICIQYKEILSQLSNTAEEIQRTKYYVNLKDITTRIVNILSDISKITQSSEIKNTSASYKQSLSFMSEALITKASLLRDLPESIVKLVSTNNLVSTLNSLPLTQDYRSKNLESLTDQIQDTLLNSIYQ |
| Q13148 | TADBP_HUMAN | MSEYIRVTEDENDEPIEIPSEDDGTVLLSTVTAQFPGACGLRYRNPVSQCMRGVRLVEGILHAPDAGWGNLVYVVNYPKDNKRKMDETDASSAVKVKRAVQKTSDLIVLGLPWKTTEQDLKEYFSTFGEVLMVQVKKDLKTGHSKGFGFVRFTEYETQVKVMSQRHMIDGRWCDCKLPNSKQSQDEPLRSRKVFVGRCTEDMTEDELREFFSQYGDVMDVFIPKPFRAFAFVTFADDQIAQSLCGEDLIIKGISVHISNAEPKHNSNRQLERSGRFGGNPGGFGNQGGFGNSRGGGAGLGNNQGSNMGGGMNFGAFSINPAMMAAAQAALQSSWGMMGMLASQQNQSGPSGNNQNQGNMQREPNQAFGSGNNSYSGSNSGAAIGWGSASNAGSGSGFNGGFGSSMDSKSSGWGM |
| Q9PVZ2 | Q9PVZ2_XENLA | MEDDTDLPPERETKDFQFRQLKKVRLFDYPADLPKQRSNLLVISNKYGLLFVGGFMGLKVFHTKDILVTVKPKENANKTVVGPQGIHVPMNSPIHHLALSSDNLTLSVCMTSAEQGSSVSFYDVRTLLNESKQNKMPFASCKLLRDPSSSVTDLQWNPTLPSMVAVCLSDGSISVLQVTDTVSVFANLPATLGVTSVCWSPKGKQLAVGKQNGTVVQYLPSLQEKKVIPCPSFYDSDNPVKVLDVLWLSTYVFTVVYAAADGSLEASPQLVIVTLPKKEDKRAERFLNFTETCYSICSERQHHFFLNYIEDWEILLAASAASVDVGVIARPPDQVGWEQWLLEDSSRAEMPMTENNDDTLPMGVALDYTCQLEVFISESQILPPVPVLLLLSTDGVLCPFHVVNLNQGVKPLTTSPEQLSLDGEREMKVVGGTAVSTPPAPLTSVSAPAPPASAAPRSAAPPPYPFGLSTASSGAPTPVLNPPASLAPAATPTKTTSQPAAAATSIFQPAGPAAGSLQPPSLPAFSFSSANNAANASAPSSFPFGAAMVSSNTAKVSAPPAMSFQPAMGTRPFSLATPVTVQAATAPGFTPTPSTVKVNLKDKFNASDTPPPATISSAAALSFTPTSKPNATVPVKSQPTVIPSQASVQPNRPFAVEAPQAPSSVSIASVQKTVRVNPPATKITPQPQRSVALENQAKVTKESDSILNGIREEIAHFQKELDDLKARTSRACFQVGSEEEKRQLRTESDGLHSFFLEIKETTESLRGEFSAMKIKNLEGFASIEDVQQRNKLKQDPKYLQLLYKKPLDPKSETQMQEIRRLNQYVKNAVQDVNDVLDLEWDQYLEEKQKKKGIIIPERETLFNSLANHQEIINQQRPKLEQLVENLQKLRLYNQISQWNVPDSSTKSFDVELENMQKTLSQTAIDTQTKPQAKLPAKISPVKQSQLRNFLSKRKTPPVRSLAPANLSRSAFLAPSFFEDLDDVSSTSSLSDMADNDNRNPPPKEIERQETPPPESTPVRVPKHAPVARTTSVQPGLGTASLPFQSGLHPATSTPVAPSQSIRVIPQGADSTMLATKTVKHGAPNITAAQKAAVAAMRRQTASQIPAASLTESTLQTVPQVVNVKELKNNGPGPTIPTVIGPTVPQSAAQVIHQVLATVGSVSARQAAPAAPLKNPPASASSIAPQTWQGSAPNKPAAQAIPKSDPSASQAPAPSVSQVNKPVSFSPAAGGFSFSNVTSAPVTSALGSSSAGCAATARDSNQASSYMFGGTGKSLGSEGSFSFASLKPASSSSSSSVVEPTMSKPSVVTAASTTATVTSTTAASSKPGEGLFQGFSGGETLGSFSGLRVGQADEASKVEVAKTPTAAQPVKLPSNPVLFSFAGAPQPAKVGEAPSTTSSTSASLFGNVQLASAGSTASAFTQSGSKPAFTFGIPQSTSTTAGASSAIPASFQSLLVSAAPATTTPSAPINSGLDVKQPIKPLSEPADSSSSQQQTLTTQSAAEQVPTVTPAATTATALPPPVPTIPSTAEAKIEGAAAPAIPASVISSQTVPFTSTVLASQTPLASTPAGGPTSQVPVLVTTAPPVTTESAQTVSLTGQPVAGSSAFAQSTVTAASTPVFGQALASGAAPSPFAQPTSSSVSTSANSSTGFGTSAFGATGGNGGFGQPSFGQAPLWKGPATSQSTLPFSQPTFGTQPAFGQPAASTATSSAGSLFGCTSSASSFSFGQASNTSGTSTSGVLFGQSSAPVFGQSAAFPQAAPAFGSASVSTTTTASFGFGQPAGFASGTSGSLFNPSQSGSTSVFGQQPASSSGGLFGAGSGGASTVGLFSGLGAKPSQEAANKNPFGSPGSSGFGSAGASNSSNLFGNSGAKAFGFGGTSFGDKPSATFSAGGSVASQGFSFNSPTKTGGFGAAPVFGSPPTFGGSPGFGGSPAFGTAAAFSNTLGSTGGKVFGEGTSAATTGGFGFGSNSSTAAFGSLATQNTPTFGSISQQSPGFGGQSSGFSGFGAGPGAAAGNTGGFGFGVSNPTSPGFGCWRS |
| G5EEH9 | NUP98_CAEEL | MFGQNKSFGSSSFGGGSSGSGLFGQNNQNNQNKGLFGQPANNSGTTGLFGAAQNKPAGSIFGAASNTSSIFGSPQQPQNNQSSLFGGGQNNANRSIFGSTSSAAPASSSLFGNNANNTGTSSIFGSNNNAPSGGGLFGASTVSGTTVKFEPPISSDTMMRNGTTQTISTKHMCISAMSKYDGKSIEELRVEDYIANRKAPGTGTTSTGGGLFGASNTTNQAGSSGLFGSSNAQQKTSLFGGASTSSPFGGNTSTANTGSSLFGNNNANTSAASGSLFGAKPAGSSLFGSTATTGASTFGQTTGSSLFGNQQPQTNTGGSLFGNTQNQNQSGSLFGNTGTTGTGLFGQAQQQPQQQSSGFSFGGAPAATNAFGQPAAANTGGSLFGNTSTANTGSSLFGAKPATSTGFTFGATQPTTTNAFGSTNTGGGLFGNNAAKPGGLFGNTTNTGTGGGLFGSQPQASSGGLFGSNTQATQPLNTGFGNLAQPQIVMQQQVAPVPVIGVTADVLQMQANMKSLKSQLTNAPYGDSPLLKYNANPEIDGKSSPASTQRQLRFLAAKKGALSSSSDAQDSSFIIPPISKVMSDLSPAVTRSADVTKDLNYTSKEAPPSLARGLRNSTFNPNMSLTNRSVHESSALDKTIDSALDASMNGTSNRLGVRGSVRRSNLKQLDMSLLADSSRVGRESRVADPDALPRISESERRQDVVTSTPAVDPVQAVIQRHNDRNRDPPSLNLDTTCDEHTGLEPVSAATSSAASVVSTPSEETVNVNSAAGVKLTKPDYFSLPTINEMKNMIKNGRVVLEDGLTVGRSSYGSVYWPGRVELKDVALDEIVVFRHREVTVYPNEEEKAPEGQELNRPAEVTLERVWYTDKKTKKEVRDVVKLSEIGWREHLERQTIRMGAAFKDFRAETGSWVFRVDHFSKYGLADDDEPMDGSPPQQALQASSPLQVIDMNTSARDVNNQVQRKKVHKATDAHHQEIILERVPAPAALGDVVPIIRRVNRKGLGGGTLDDSREESCIGNMTTEFNESGHDSIIEEGQQPEKKPKLELLADLEYESSRFIRNLQELKVMPKANDPAHRFHGGGHSAKMIGYGKSKLIDIGIVKGRSSHVGWSETGCLVWSAQPRHNQVLFGTIDRTSDVNENTLISMLDVNVHVSETSRKGPSSQSNSVKSSLTSNFVTYSDSYSSMFAKYIDVAQAGGYDGHVSVWKLISALFPYERREGWSFERGEEIGEWLRTEAVKSVPDDRSADTSSNGVWNQLCLGDIDKAFQIAIDNNQPQLATMLQTSAVCPEATVHCFKAQLDNWKKCETLHLIPKETLKCYVLMSGLSHYEWDQDGKNHSINCLDGLNWIQALGLHVWYLRAWTGLEESYDAYQKDVNAGRAASNRGDLPGELIKLACESQHSVEVVLDCAAGENPNDYFLQWHVWSLLYSVGYRTMSKTSETRLHRNYSSQLEASSLSKYALFVLQHIDDDEERSTAVRSLLDRIARFTDNDMFDSISEQFDIPSEWIADAQFSIAKSVDDSTQLFELAVAAKNYLEICRLFVDDIAPTAVVAGDHDALKAACAMVRPFENQIPEWGATGMVYTDYCRLINLIENDAEEELLQDVLESLETRLHAPTISKNSLQKLSLQTIGRVLFEYRADKNTLPEWTKLLGHRQMFKIFRDRSSWGIERFTIEFD |
| P52948 | NUP98_HUMAN | MFNKSFGTPFGGGTGGFGTTSTFGQNTGFGTTSGGAFGTSAFGSSNNTGGLFGNSQTKPGGLFGTSSFSQPATSTSTGFGFGTSTGTANTLFGTASTGTSLFSSQNNAFAQNKPTGFGNFGTSTSSGGLFGTTNTTSNPFGSTSGSLFGPSSFTAAPTGTTIKFNPPTGTDTMVKAGVSTNISTKHQCITAMKEYESKSLEELRLEDYQANRKGPQNQVGAGTTTGLFGSSPATSSATGLFSSSTTNSGFAYGQNKTAFGTSTTGFGTNPGGLFGQQNQQTTSLFSKPFGQATTTQNTGFSFGNTSTIGQPSTNTMGLFGVTQASQPGGLFGTATNTSTGTAFGTGTGLFGQTNTGFGAVGSTLFGNNKLTTFGSSTTSAPSFGTTSGGLFGNKPTLTLGTNTNTSNFGFGTNTSGNSIFGSKPAPGTLGTGLGAGFGTALGAGQASLFGNNQPKIGGPLGTGAFGAPGFNTTTATLGFGAPQAPVALTDPNASAAQQAVLQQHINSLTYSPFGDSPLFRNPMSDPKKKEERLKPTNPAAQKALTTPTHYKLTPRPATRVRPKALQTTGTAKSHLFDGLDDDEPSLANGAFMPKKSIKKLVLKNLNNSNLFSPVNRDSENLASPSEYPENGERFSFLSKPVDENHQQDGDEDSLVSHFYTNPIAKPIPQTPESAGNKHSNSNSVDDTIVALNMRAALRNGLEGSSEETSFHDESLQDDREEIENNSYHMHPAGIILTKVGYYTIPSMDDLAKITNEKGECIVSDFTIGRKGYGSIYFEGDVNLTNLNLDDIVHIRRKEVVVYLDDNQKPPVGEGLNRKAEVTLDGVWPTDKTSRCLIKSPDRLADINYEGRLEAVSRKQGAQFKEYRPETGSWVFKVSHFSKYGLQDSDEEEEEHPSKTSTKKLKTAPLPPASQTTPLQMALNGKPAPPPQSQSPEVEQLGRVVELDSDMVDITQEPVLDTMLEESMPEDQEPVSASTHIASSLGINPHVLQIMKASLLTDEEDVDMALDQRFSRLPSKADTSQEICSPRLPISASHSSKTRSLVGGLLQSKFTSGAFLSPSVSVQECRTPRAASLMNIPSTSSWSVPPPLTSVFTMPSPAPEVPLKTVGTRRQLGLVPREKSVTYGKGKLLMDMALFMGRSFRVGWGPNWTLANSGEQLNGSHELENHQIADSMEFGFLPNPVAVKPLTESPFKVHLEKLSLRQRKPDEDMKLYQTPLELKLKHSTVHVDELCPLIVPNLGVAVIHDYADWVKEASGDLPEAQIVKHWSLTWTLCEALWGHLKELDSQLNEPREYIQILERRRAFSRWLSCTATPQIEEEVSLTQKNSPVEAVFSYLTGKRISEACSLAQQSGDHRLALLLSQFVGSQSVRELLTMQLVDWHQLQADSFIQDERLRIFALLAGKPVWQLSEKKQINVCSQLDWKRSLAIHLWYLLPPTASISRALSMYEEAFQNTSDSDRYACSPLPSYLEGSGCVIAEEQNSQTPLRDVCFHLLKLYSDRHYDLNQLLEPRSITADPLDYRLSWHLWEVLRALNYTHLSAQCEGVLQASYAGQLESEGLWEWAIFVLLHIDNSGIREKAVRELLTRHCQLLETPESWAKETFLTQKLRVPAKWIHEAKAVRAHMESDKHLEALCLFKAEHWNRCHKLIIRHLASDAIINENYDYLKGFLEDLAPPERSSLIQDWETSGLVYLDYIRVIEMLRHIQQVDCSGNDLEQLHIKVTSLCSRIEQIQCYSAKDRLAQSDMAKRVANLLRVVLSLHHPPDRTSDSTPDPQRVPLRLLAPHIGRLPMPEDYAMDELRSLTQSYLRELAVGSL |
| P49790 | NU153_HUMAN | MASGAGGVGGGGGGKIRTRRCHQGPIKPYQQGRQQHQGILSRVTESVKNIVPGWLQRYFNKNEDVCSCSTDTSEVPRWPENKEDHLVYADEESSNITDGRITPEPAVSNTEEPSTTSTASNYPDVLTRPSLHRSHLNFSMLESPALHCQPSTSSAFPIGSSGFSLVKEIKDSTSQHDDDNISTTSGFSSRASDKDITVSKNTSLPPLWSPEAERSHSLSQHTATSSKKPAFNLSAFGTLSPSLGNSSILKTSQLGDSPFYPGKTTYGGAAAAVRQSKLRNTPYQAPVRRQMKAKQLSAQSYGVTSSTARRILQSLEKMSSPLADAKRIPSIVSSPLNSPLDRSGIDITDFQAKREKVDSQYPPVQRLMTPKPVSIATNRSVYFKPSLTPSGEFRKTNQRIDNKCSTGYEKNMTPGQNREQRESGFSYPNFSLPAANGLSSGVGGGGGKMRRERTRFVASKPLEEEEMEVPVLPKISLPITSSSLPTFNFSSPEITTSSPSPINSSQALTNKVQMTSPSSTGSPMFKFSSPIVKSTEANVLPPSSIGFTFSVPVAKTAELSGSSSTLEPIISSSAHHVTTVNSTNCKKTPPEDCEGPFRPAEILKEGSVLDILKSPGFASPKIDSVAAQPTATSPVVYTRPAISSFSSSGIGFGESLKAGSSWQCDTCLLQNKVTDNKCIACQAAKLSPRDTAKQTGIETPNKSGKTTLSASGTGFGDKFKPVIGTWDCDTCLVQNKPEAIKCVACETPKPGTCVKRALTLTVVSESAETMTASSSSCTVTTGTLGFGDKFKRPIGSWECSVCCVSNNAEDNKCVSCMSEKPGSSVPASSSSTVPVSLPSGGSLGLEKFKKPEGSWDCELCLVQNKADSTKCLACESAKPGTKSGFKGFDTSSSSSNSAASSSFKFGVSSSSSGPSQTLTSTGNFKFGDQGGFKIGVSSDSGSINPMSEGFKFSKPIGDFKFGVSSESKPEEVKKDSKNDNFKFGLSSGLSNPVSLTPFQFGVSNLGQEEKKEELPKSSSAGFSFGTGVINSTPAPANTIVTSENKSSFNLGTIETKSASVAPFTCKTSEAKKEEMPATKGGFSFGNVEPASLPSASVFVLGRTEEKQQEPVTSTSLVFGKKADNEEPKCQPVFSFGNSEQTKDENSSKSTFSFSMTKPSEKESEQPAKATFAFGAQTSTTADQGAAKPVFSFLNNSSSSSSTPATSAGGGIFGSSTSSSNPPVATFVFGQSSNPVSSSAFGNTAESSTSQSLLFSQDSKLATTSSTGTAVTPFVFGPGASSNNTTTSGFGFGATTTSSSAGSSFVFGTGPSAPSASPAFGANQTPTFGQSQGASQPNPPGFGSISSSTALFPTGSQPAPPTFGTVSSSSQPPVFGQQPSQSAFGSGTTPNSSSAFQFGSSTTNFNFTNNSPSGVFTFGANSSTPAASAQPSGSGGFPFNQSPAAFTVGSNGKNVFSSSGTSFSGRKIKTAVRRRK |
| Q5EAX5 | Q5EAX5_XENLA | MASGFSFGTAAASTTTLNPTAAAPFSFGATPAASNTGTTGGLGFGAFNAAATPATTTATTGLGGGLFGAKPAAGFTLGGANTATATTTAASTGFSVGFNKPAGSATPFSLPVTSTSSGGLSLASALTSTPATGPSPFTLNLGSTPATTTAAATGLSLGGTLTGLGGSLFQNTNPSATGLGQSTLGQSTLGQSTLGQSLLGQSLLGQSLLGQSTLGQSTLGQSLLGQSLLGLGLNLGAVAPVSQVTTHEGLGGLDFSSSSDKKSDKAGTRPEDSKALKDENLPQLLCQDVENFQKFVKEQKQVQEEISRMSSKAMLKVQEDIKALKQLLSVASSGLQRNALAIDKLKIETAEELKNAEIALRTQKTPPGLQHENTAPADYFHTLVQQFEVQLQQYRQQIEELENHLATQSNTLHLSPQDLSMAMQKLYQTFVALAAQLQAVNENFKMLKEQYLGYRKAFLGDSTDVFEARRAEAKKWQNAPRVTTGPTPFSNIPNAAAVAMAATLTQQQQPTTGFGSSSAFGGNTSGSSSFGFGTANKPSGSLSAGFGSTSTSGFNFSNPGINASAGLTFGVSNPSSTSFGTGQLLQLKKPPAGNKRGKR |
| Q9JMD0-3 | ZN207_MOUSE | MGRKKKKQLKPWCWYCNRDFDDEKILIQHQKAKHFKCHICHKKLYTGPGLAIHCMQVHKETIDAVPNAIPGRTDIELEIYGMEGIPEKDMDERRRLLEQKTQESQKKKQQDDSDEYDDDESAASTSFQPQPVQPQQGYIPPMAQPGLPPVPGAPGMPPGIPPLMPGVPPLMPGMPPVMPGMPPGLHHQRKYTQSFCGENIMMPMGGMMPPGPGIPPLMPGMPPGMPPPVPRPGIPPMTQAQAVSAPGILNRPPAPTAAVPAPQPPVTKPLFPSAGQAQAAVQGPVGTDFKPLNSTPAATTTEPPKPTFPAYTQSTASTTSTTNSTAAKPAASITSKPATLTTTSATSKLIHPDEDISLEERRAQLPKYQRNLPRPGQTPIGNPPVGPIGGMMPPQPGLPQQQAMRPPMPPHGQYGGHHQGMPGYLPGAMPPYGQGPPMVPPYQGGPPRPPMGMRPPVMSQGGRY |
| C3XWA2 | C3XWA2_BRAFL | MFGQQKTPFGGTTGFGTGAFGTSSFGATQTPATGGLFGGTATNTGTGLFGGTSFGTPSTSTSTFGGFGTSTTQTGGGLFGTSTSTAGTGLFATPQQQTAPFGAANKTGFGGFGTQTSTAATGTGLFGATQQTPSLFGGGQTTSTGLFGAVGGIAAGTNGTTVKFNPVSGSDTMMKNGVSQNIRTAHQCITAMKEYETKSLEELRVEDYLANRKGGSTGTTAMFGATATPQTGGGLFGNTATTTSTTGFTFGKAAFGTGQTQAKGATGFGTTSTGTGLFGQTQTTQAGGLFASPFGGTATTTTPSTGFSFGQTNTGTGLFGQTQQKTGLFGQPTTQTTGLFGTPSTTTTTGFGTTFGTGTFGTQNQAGGLFGANKAPTFGATTTTSTTGGLFGNTATNTGGLFGQNKPGLTLGLGTGFGTGAFGTTTTSTGTSLFGPKPTNTFGAGLGTGLGAGLGTLGTGSIFGNTGLGAGIGTGTTGLNLTQPAAALGTDAAAQLAQQAQIQQQLQALSNSPFGDSPLFRNNVADPAKRAELLKPTSLAAQKALTTPSHYKISPRPTAKIKPKPLHSLVAGKSQLFEGLDDEDTSLSNDTFVPRRSVKKLIIRNKASPTASELDAGLGEEDDLLAPPAYPSDTFLVEDKRTSESEKENDPMENLYSNPVRKPIPETPANKSSPAPDDSIIALNVRQRSCSPGQDSMGEPTSDDQGEEEVEREPHPAGIILTKNEYYTEPSLDELANMVDENGDCWVENFVIGREGYGSVFFPGLTNVANLNLDETVHIRRKEITVYPDDSTKPPEGDGLNKKAEVTLHCTWPMDKTAHIPIKSPDRLKKMGYQEKIEVATSKIGARFLEYRPDTGSWVFQVPHFSKYGLDDSEDEEELVGQDQKKMKMQQQTDLQKQQLQKENQPPSLNKQQPITERVKSEQPIMEQMQDDSDMADISQEPIPDLAPNSSGDQDGLEGLSEEEVVPSSHRLATHLGMSAHRMQVMKASFFGDEEEGEGMDYKPVFGGMSGRSSLRGSAPPSGAASPLPHRMAESKMDRARLGSLGSPVRGLGGMSPSHSPRTRIPSGGFSPKYSPKVEQSGLFPSLSKPAQYPVFERNQGLLLPSGLSSEIERPRKIVGSQRQPMLLPWRETVTNEKQSLVADAALMMGRSFKVGWGPNWTLVRCAGADREGEAKERKKETSIPFSILPKSTPRLAKLGTSSPFSVVMERVDVAPHFVVQDKVTVANHVSHLEVELETSLCSTEDHPCPVFVPTPGVNALHQHAEVAGRNRENTGVRHPERESADHASLVLSLCVALWGDLPDQDREDGEDGLDRTSYAYHMARREAVSHWLMNQAQDTIEQEVQDSKFKEGGHTDAIFSLLSGRQISQACSLAQQSGDHRLALLLAQASGSHFPRELVGKQLSDWEELKADRFISDSRLRVYALLAGKPVWPATNGEINTCENLDWKRALAVHLWYLCTPSATVSEALHLYCKAFTKNNSDYGEYANPPLPPYLERSADRSGDQEDRYVIRDMCYHLLQLYADRSHQLDRLLAPTTSTPNQLDYRLSWLLCQALQALDYTHLSEHHLNTIHAGYIAQLESLGLWEWAVFVALHITDNSRRELVTKELLCRHCSLSEEEAYVQKEEFLQEKLRVPVVWIHEAKALRAQYEGKSHDEAWHLLKAEKWNASHRIILKHLAADAIINEDYEYLKEFLEELSPPDRSSTIQDWNFGGRVFLDYVLINNALVEMAKGNSSSYDLERLHPQVTSLCSRIENIPCLTPKDRLCQSEMAKKAASFLRLVLEGQNKPPTVDGEYLVPSYQLAPHITKLPMPEDYALQELRALTHSYMMELTT |
| J7I6Y1 | J7I6Y1_XENTR | MFNKTFGSPFGTGNGAFGATSTFGQTTGFGTTPATAFGSAGFGTNTSTGGLFGNTQTKPGGLFGSTTFNQPATSSSSSGFGFGASTGTTNSLFGSTNTGSGLFATQSNAFGQAKPTTFGNFGTSTSTGGLFGNTNTANPFGGTSASLFGASTFSAAPTGTTIKFNPPSGTDTMAKGGVTTNISTKHQCITAMKEYESKSLEELRLEDYQANRKGPQNPVGAPTGTGLFGTSAATSSASTGIFGSTAANNSFSFAGNKTTFGTAGTGAFGGNTGGLFGQPANQPAASLFNKPFGNATTTQSTGFSFGNTSTLGQPQTSTMGLFGANQPTQSGGLFGTTTNTNATGAFGAGTSLFGQPNPAPFGTGSTLFGNKPAGFGTTTTSAPAFGTTTGGLFGNKPTLTLGTNTNTSNFGFGSNTAGTSLFGNKTATGTIGPSLGTGFGTALNPGQTSLFGSNQPKLTGTLGTGAFGNAGFNSTSAGLGFGAPQAPVAALSDPGASAAHQMFLQQQYNALRYSPFPDSPLFRNPISDPKKKEELLKPTNPAAQKAVLTPTHYKLTPRPAARVRPKALQNSGAAKSQLFDALDDDEPVLSSGVFMPKKSIKKLDLKYLKNSSSLFGQENRDVDDMVPPTPEAPERSVENHHGDEENDEEAATTYPSPFSRPLPQSQEKKQVNHMDDTIVALNMRKSGRIGLEHSSEDASFNEDSFRETEILDASPHPAGIILTRDSYYTIPSMEELARSVDENGECIVNGFTIGREGFGSIYFEGIVNLTNLDLDSIVHIRRKEVIVYVDDQNKPPLGEGLNRPAQVTLDEVWPIDKTSRCMITSPERLSEMNYKSKLENASRKQGAQFVDYRPESGSWVFKVNHFSKYGLQDSDEEDDQLNSAEAKKLKTAPVPPQGKQPPLQQATLPGKVTPPPQSPAVDQLDRVMELDSDMADITQDQDLDSVAEEQDISEEQEPLSASSHIASSLGINPHALQVMKASLLLEEEDGEIMSRFSSFPSSMDPYPDVRSPRLFPSSHAKRPSSIGLLQSKFSAPSMSRLSETVQGSHSPKIHPAAPWSVPAPLAPSFIMPGPAPDTHLRTVGTRRQQELVPLEKSVTHGRGTLMIDMGLFMGRSFRVGWGPNWTLVHNGDKLSERLNAEEDRDMDTIDYGFLPKPTSAKSLTESPFKVHVEKLSLEQKTKDLQSYLLPLEIELKNSTVDKSGPCPHFRPNPGVTAIHDYAGWVRNFSSEAAEVEAVVKQWGLTWTLCESLWGQLKELEASLDEPNEYVKNLERRKAFSHWLAQTAQERIEEEVSLYGPERHIEAVFSYLTGGRISDACRLAQKSGDHRLSLLLSQMVGSQEVRDLITLQLVDWNKLQVDHYIQEERLRVFCLLSGTPVWRSSDNRSINVCSQLDWKRTLGIHLWYMLPPTATVAQALHMYEQAFQEQEGGEPYACYPLPPYLEDCGFSFGDDPSAKFCSLQRDVCVHLLKLYSERQYDLCQLLDPSSATPDPLDYRLSWHMWMVLQALNYTHLSGHRQGMLHASYAAQLENVGLWEWAIFVLLHIQDPHVREAAVRELLNRHCVVHDSPESLAKENFLIQRLCLPAQWIHKAKAVRSRRDGDKHKEALYLLKSHQWNQCHKLVTRHLAADAVINENYRYLRGFLGELARPEHCKHIQDWETAGKVYLDYISVIEMLNQIRQDECSGGELEKLHTKVMSLCKWVELIQCYSAKGRLAQSEMAKRVANILRVVLSLQQPPESMSDSSSEPRVPLRLLAPHIGRLPMPEDYALEELRGLTQSYLRELICGS |
| Q9VCH5 | NUP98_DROME | MFGGAKPSFGATPAATSFGGFSGTTTTTPFGQSAFGKPAAPAFGNTSTFAAQPAQQSLFGAAATPAQPAGGLFGANTSTGFGSTATAQPTAFGAFSQPQQTSNIFGSTQTAASTSLFGQSTLPAFGAAKPTMTAFGQTAAAQPTGSLFGQPAAATSTTGFGGFGTSAPTTTNVFGSGTASAFAQPQATAVGASGVNTGTAVAKYQPTIGTDTLMKSGQANSVNTKQHCITAMKEFEGKSLEELRLEDYMCGRKGPQAGNAPGAFGFGAQVTQPAQPASGGLFGSTAQPSTGLFGQTVTENKSMFGTTAFGQQPATNNAFGAATQQNNFLQKPFGATTTTPFAAPAADASNPFGAKPAFGQGGSLFGQAPATSAAPAFGQTNTGFGGFGTTAGATQQSTLFGATPAADPNKSAFGLGTAASAATTGFGFGAPATSTAGGGLFGNKPATSFAAPTFGATSTASTPFSNFGLNTSTAATGGGLFNSGLNKPATSGFGGFGATSAAPLNFNAGNTGGSLFGNTAKPGGGLFGGGTTTLGGTGAAPTGGLFGGGTTSFGGVGGSLGGGGFGMGTNNSLTGGIMGAQPTLGIMTPSHQPIHQQILARVTSPYGDSPIFKDLKLSSEADATRATNPAAQQAVLDLTSNQYKISTSNNPAPMKVKALGSTLNRKSLFDGLEEFDASVEGFNLKPSAKRLVIKPKVKSVEGGNPSSSIGSAPNTPQSRPKGATPNKERESFSGAIPSEPLPPAGNSPGATNGRESQDNGRRESWLHPNNLEKVRQHNIQTGMDQGSPHNSTLNELVPRKPLDTYRPSSTVRLSVSTIPENPFEDQSSTIARRETFTSQQANESVLSNRSNEAEDSAANQSRLAIEAAAAEAADDESHPTGIVLRRVGYYTIPSLDDLRSYLAEDGSCVVPNFTVGREGYGNVFFGKEMDVAGLNLDEIVHFRNKEIIIYPDDENKPPIGQGLNRDAQVTLDQVWPLDKTKHEAIKDPQRLLEMDWEGKLRRVCDKNDTRFIEYRPETGSWVFRVKHFSKYGLGDSDEEDELPTDPKKAKIATLEAQQRANAEKMTLNSLRQAQKISEDAARNLDPKALVAGVASGFRPMDDTAEFLLMDKTQFFQAGGNSDFSMFDPPRQRPTITSPTAVLAQEMVGNEAHKMQLMKSSFFVEDNAPEDEPMETTGRLLRHRKFFNVEPLVWKDGASESSSQYDFEHPSPALPISSSVSEASLMCDAHYEETSSMATGSIVAAVKETKFEMPVTKAFKFVCKPKVAPIKLRATTVPLPRSIAYEMRDNWIADLGFYKGRSFKLSFGPQNSLVLPSTYNNMQNLKEFTGPSLPVSMVFAPRSATDLSPSVMQLVEFNMVKGNEGFRESIIPHLEVQLNDCLSVNVEGSECPCIHPDSGTKLVSKHFSESLKQRNAGLKEDYSVSVWSLLFALWGDHDELVDLEKNSHYMVMCRRNLLSEWLENTLLGKDLLSKKVSTHSYLEHMLDLLSCHRVNEACELAFSYDDANLALVLSQLSSGAVFRLLMEEQLFAWQQSKSDKYIDLERLKMYMLAAGAPMMQSSHGAINLLENKNWLTALALQLWYFTAPTSSITDALNAYNDAFQAEECYAEPPKPSYRDAPTDTKKPVYDLRYHLLQLHSKRMHSLEETLNPITHTADAMDFRLSWLLLQTLRALGYRHCSPLTEARLSVDFASQLENEGLWQWGIFVLLHIKQQTQRERAVQQMLQRNVSVSAKVALYAEERFIVEELGIPMSWVDYAKAVKAGASGKHHLQAKYLLKAKHFATAHDVIFQHIAPDAIINGKMKYLHSLLIQFEDTEGSSIRVPNWANQGQIFLDFIDISAKFKQIRSVTNIADINARWENLKPQLSELCSRISLLPCPTSKHRLCQSEISQSLSCLVHGMCIVCPEMESSTVLKVALERLPLPQEFASKELRIWLEELLDKIQNEPPFSERQQPTMMEI |
| K9ZRR1 | K9ZRR1_XENTR | MAAAGGGGGAGESGTGGKIRSRRYHLSSGRTPYSKSRQRQGIISRVTDAVKSIVPGWLQNYFNKQEEERGRAHNASELIVEDTEGRQNNTEHHIYVDDEGNSASGRVTPEPIINVDEEVPSTSQSAINDTDALTRPSLHRANLNFNLFDSPAMNCQPTTSTFPIGTSGFSLVKEIKDSTSQHDDDNISTTSGFSSRASDKDMAVSKNVGVPPLWSPEVDRSQSLSHNSSMTSKKPTFNLSAFSSLSPSLGNASFVNSQLGDSPFYPGKTTYQGAAAVRSSRVRSNPYQAPLRRQVKAKPANAQQYGVTSSTARRILQSLEKMSSPLADAKRIPSNSLSHTPEKSLMDIAENPSKRKKVESPFPPVQKLVTPKSISVSTNRSLYIKPSLTPSAVSNTSSRRVQPDKHNESRENNVQTVQSTSHSFSYPKFSTPTSNELLSRGGGKMMRQKGSHYSTKPAEETSGLTSSSPLFTFSSPIVKSTESNAQSPGSSVDFTFSVPTVKVSSATTSIDSKVSAVSTTAKTHIAVNNSSAKDSDEQVGFCKPAKTLKEGSVLDILRSPGFSSSPSQQTSASTPNRSTSTFSKTVGNAFSPVKVTLGVGGKQALGLWQCSACCHENMASDSNCTACCAAKPQPAETNKKLPASPQHSNTKTTVPLSSVQGLGDKFKLPAGTWDCDTCLVQNKAEVTKCVACETPKPGTGMKANLLLPPATKALNSAPTPSAFVSSSGSITNEEKPEGSWDCTGCRMQNKTENNTCVACKADKPGAIKSLSTAAPSGLLGLLDHFKKPAGSWDCDVCLIQNKPEATKCVACESAKPGTKPELKGTFGTVQTSAAPVSLGLGLLDQFKKSAGSWDCEVCLVENKPEANKCIACESAKPGTKAELKGFGTSTLSAGTTAPTFKFGVQPSDSAGELKSGASTDSTSGFSFAKPIGDFKFGLASASATTEETGKKSFTFGTSTSNQASAGFKFGVASSAQTNQDTSGGFTFGSVSSTVSFSPAATYSGSTGLQVPAADDDSSRASAAGLKSAEEKKPEAPAVTAFSFGKTDQNKETVSTSFIFGKKDEKTDSAPTGNSFGFGLKKDGEEPKQFLFGKPEPTKEDSTSSTASAGFAFRVSNPTEKKDVEQPVKSVFAFGSQTSTTDAGASKQPFSFLTGVSSTSASSSASGVSSSVFGSVAQSSTPANPSNVFGSATSSNPPAVSSGVFGNLNPSNAPASSSTLFGNVAPSSTPSGSSSLFGTANPSSTPASSSSLFGTAAKLSAPVGSGGVFNSAAPVPPASTSSSVFGSAAPANTSANSANIFGSAGGTSGAPGTFVFGQPASTTSTVFGNSSESKSTFAFSGQETKPVTSAITSATPFVFGAESASTTPAAPGFNFGRTNTSNVTGTSSSPFIFGGGPTASASPSLTAHANPVPAFGQSANSSTAPAFGSSTSVFPAGNSQQVPAFGSSTAQPPVFGQQAAQPSFGSSAAPSAGSGFQFGNNTNFNFTTPNSSGGVFTFGANAGSTPQPPAPGFMFNAAASGFNVGTNGRSTPASSISNRKIKTARRRK |
| Q96PK6 | RBM14_HUMAN | MKIFVGNVDGADTTPEELAALFAPYGTVMSCAVMKQFAFVHMRENAGALRAIEALHGHELRPGRALVVEMSRPRPLNTWKIFVGNVSAACTSQELRSLFERRGRVIECDVVKDYAFVHMEKEADAKAAIAQLNGKEVKGKRINVELSTKGQKKGPGLAVQSGDKTKKPGAGDTAFPGTGGFSATFDYQQAFGNSTGGFDGQARQPTPPFFGRDRSPLRRSPPRASYVAPLTAQPATYRAQPSVSLGAAYRAQPSASLGVGYRTQPMTAQAASYRAQPSVSLGAPYRGQLASPSSQSAAASSLGPYGGAQPSASALSSYGGQAAAASSLNSYGAQGSSLASYGNQPSSYGAQAASSYGVRAAASSYNTQGAASSLGSYGAQAASYGAQSAASSLAYGAQAASYNAQPSASYNAQSAPYAAQQAASYSSQPAAYVAQPATAAAYASQPAAYAAQATTPMAGSYGAQPVVQTQLNSYGAQASMGLSGSYGAQSAAAATGSYGAAAAYGAQPSATLAAPYRTQSSASLAASYAAQQHPQAAASYRGQPGNAYDGAGQPSAAYLSMSQGAVANANSTPPPYERTRLSPPRASYDDPYKKAVAMSKRYGSDRRLAELSDYRRLSESQLSFRRSPTKSSLDYRRLPDAHSDYARYSGSYNDYLRAAQMHSGYQRRM |
| K9ZTJ6 | K9ZTJ6_XENLA | MAFNFGATTGTPANQGTTGFSLGTFTPKTTTSGFGFGTTTTTAPTGFGGGFGGFGATTTASTGPAFSFTTPANTTSGLFGATQNKGFGFGTGFGSTTTSTGLGTGLGTGLGFTGFNTSQQQQQQSVLGAGLFNQSFQSTPQSNQLINTASALSAPTLLGDERDAILAKWNQLQAFWGTGKGFFMNNTPPVEFTQENPFCRFKAVGFSYIPNNKDEDGLISLIFNKKESDIRGQQQQLVESLHKVLGGHQTLTVNVEGVKTKADNQTEVIIYVVERSPNGTSRRVGASALFSYFEQAHIKANMQQLGVTGAMAQTELSPVQIKQLIQNPLSGVDPIIWEQAKVDNPDPERLIPVPMIGFKELLRRLEVQDQMTKQHQSRLDIISEDIGELQKNQTTTMAKIGQYKRKLMELSHRVLQVLIKQEIQRKSGFAIQAEEEQLRVQLDTIQSELNAPTQFKGRLNELMSQIRMQNHFGAVRSEEKYYVDADLLREIKQHLKQQQEGVSHLISIIKDNHEDIKLIEQGLNDNLHMRTGFLS |
| Q7ZXV8 | ZN207_XENLA | MGRKKKKQLKPWCWYCNRDFDDEKILIQHQKAKHFKCHICHKKLYTGPGLAIHCMQVHKETIDAVPNAIPGRTDIELEIYGMEGIPEKDMEERRRILEQKTQVDGQKKKTNQDDSDYDDDDDTAPSTSFQQMQTQQAFMPTMGQPGIPGLPGAPGMPPGITSLMPAVPPLISGIPHVMAGMHPHGMMSMGGMMHPHRPGIPPMMAGLPPGVPPPGLRPGIPPVTQAQPALSQAVVSRLPVPSTSAPALQSVPKPLFPSAGQAQAHISGPVGTDFKPLNNIPATTAEHPKPTFPAYTQSTMSTTSTTNSTASKPSTSITSKPATLTTTSATSKLVHPDEDISLEEKRAQLPKYQRNLPRPGQAPISNMGSTAVGPLGAMMAPRPGLPPQQHGMRHPLPPHGQYGAPLQGMAGYHPGTMPPFGQGPPMVPPFQGGPPRPLMGIRPPVMSQGGRY |
| Q91349 | Q91349_XENLA | MSGFNFGAASAGGFSFGNPKSTTTTAPTGFSFGAATAAPSGGFSFGTATPTPASTTGQTSGLFSFSNPAPSLAPTSGFSFGAQVTSTPAPSSGGLAFGANTSKLNSGVGNQPAGGTTQTSQPMGGFSFGAATTQTQPSATSVGGFSFAGGVGSTSTNVFAQPAASTGITLQSAVSTAAAPTATTSQPTSTFSFGTQPQAAPALNFGLLSSSSVLSTASTPAAAQPVAPTTGLSLNFGKPADTSAAVTSTGSTTTNTPSLSSLLGTSGPSLFSSVATSTVPSVVSTVASGLSLTSTATSTGFGMKTLASSAVPTGTLATSTASLGVKAPLAGTIVQANAVGSAAATGISTATAMTYAQLENLINKWSLELEDQEKHFLQQATQVNAWDRTLMQNGERITTLHREMEKVKLDQKRLDQELDFILSQQKELEDLLTPLEESVKEQSGTIYLQHADEEREKTYKLAENIDAQLKRMAQDLKEVIEHLNTSAGPGDASNPLQQICKILNAHMDSLQWIDQNSALLQRKVEQVTKECESRRKEQERGFSIAFD |
| Q5EWX9 | Q5EWX9_XENLA | MEGGCCWDLVKRIQPIALPLALSLVTCALAYIYNQLTVAGLLLASCWTYRSSGRRFIVWKWLTGPRLVASTKKLRNSTDWRLQSPPVSLLEGSPSPLSLNMGTYMNKQELPARGEACGPRAIKEKLSRPNPSVATPIRRLSFRDPLSSSNRTYLCARRDYPLKQEIYSKPGSLPRVSLDGQSQQRVPLTPRHFTRRTPVKISPQDLKTRFQLAPQPNVVSSPHLDFEQEPCLPSIYHRVEHETSVTHSRHLLEPEQYPASPSSHKANTFEATFSSHHVEDPCATESVLRALKECLKRKRIRDEDSEDTENKRRKENGEESKLLVNDSLCRTSKMEASRASWRSESSSMENLSQQSSISIQDMLNEVQNSKNSDGKVSPASSFNLENLSKRKIITVPGHNEITSSLSSSRYLQKKKRSLEIPRCDTPEWPIKKMRKDRLETSMSPSQETMKTQMSETQKVEVNPSPVQLHKPKGNLPWKRNVHLICPEHPEEFYRLPPGPIPGYNVTQADYDAAKEAAQQRFLRLFQEPSAPSVPSASESCSKQVSLTLQTSTSPDKSFQAVNEKPNISAANTTFTLFSSSSDAKSVTSSSSTPSPFTSATTSITNTLLQSLGSGQTKSESPFPKNSLLQILGKTEGNNSQPMFNPVFGPLGSANPSPVTAAPGLSTTTTAILKPICGDPPSQQAQTSMFKPIFEPPSSQTVPSALSSPFVFSSSSSTTSTTSFLGTTGNSVNTGKHEKPNLALTTCASNTTITSSLAPTFGITSKVEPVSLGGPASQANTSFSVIGSTSVPAISTAFGSTTSAFTAAGQTANPSFSANTAPIGVSSVSASEGQTSISTTATFSKFGVPVGQNVFSASNPFGSGTQSTMGTSTQSATNIAFSFGTSTSTQSAFGFSQNPTMLFSSSTKSNNPTNTSGFNSMGSVFGSSTVPTSSIVTPNKSLSTLGIPEKCETKNQLVTNQLSLGQGSTPAPFPSIMPTQSFVSSSTSFAPSTPTVNQSSTPGSFPSMAAVQPFTSPSSPAAGFFSHGTAPKSRTAVRHKLHPRRPHPRKK |
| P34761 | WHI3_YEAST | MQSSVYFDQTGSFASSSDNVVSSTTNTHNISPSHRSSLNLNTTSHPHEASGRGSASGELYLNDTNSPLAISSMLNTLALGSMPQDIASSNISNHDNNIKGSYSLKLSNVAKDITLRECYAIFALAEGVKSIELQKKNSSSSITSASLEDENDIFIIARFELLNLAINYAVILNSKNELFGPSFPNKTTVEIIDDTTKNLVSFPSSAIFNDTSRLNKSNSGMKRPSLLSQRSRFSFSDPFSNDSPLSQQQSQQQQQQPQQPQQHSTQKHSPQQCNQQQVNSSIPLSSQGQVIGLHSNHSHQDLSVESTIQTSDIGKSFLLRDNTEINEKIWGTSGIPSSINGYMSTPQPSTPTLEWGNTSASQHGSSFFLPSAASTAIAPTNSNTSANANASSNNGASNNGANQALSASSQQPMMQIGNTINTSLTSSNSLPPYGLMSSQSQHISNMVNTSDMNITPQKQNRFMQQPQPEHMYPVNQSNTPQKVPPARLSSSRNSHKNNSTTSLSSNITGSASISQADLSLLARIPPPANPADQNPPCNTLYVGNLPSDATEQELRQLFSGQEGFRRLSFRNKNTTSNGHSHGPMCFVEFDDVSFATRALAELYGRQLPRSTVSSKGGIRLSFSKNPLGVRGPNSRRGGSGNPNPNVNMLSSYNSNVGHIKN |
| Q5XGN1 | NUP42_XENLA | MAICNFFLQGRCRYGEKCWNEHPRGGGGGGGNRYQSQNRYQEQSRYQEQSRYPEQSRYPEQNRYQEPAGNAKGTWGASSQRYVQPSNFSKSTTWINRDSEKPSAGSFSGFGSRNVKSTAATGLPSTQNRFAALSSQDNSRDGQTDKGNILDDIMKDMEIWESSGQWMFSVYSMLKEKKNISGFTDFSPEELRLEYSVCQAEGNPLKYINAVQQLGSKWKQRILELKNPNPSIKTALLNELNSPSPDVTPGYSGQQNSAFGALSFPTSNTAPTAVTFSFKADTTTAAKPAVPNALAGSDFSAFGNKPTSAPSFGSGVAAAAASFSFAPSTISGFGSTASNSGFGAASNAAGFQGAANIAAAPAFGVASSTAPASGFGGGFGTTVNTGAKTSSVRDLFSAGTAVPVQTTLLFGQATGSLNTTASSTSLAGQPFKASTSATAVSGSFTSDNTSNPLFTPRNELSVEDLAQFEAKQFTLGKTPIKPPSADLLKVT |
| Q6DEC7 | Q6DEC7_XENLA | MAKRIADKELTDRNWDQEEEEEDAGMFSVASQEVLKTRAIKKAKRRNAGNESESGGAFKGFKGLFLVTGGSLSSFGNGSPAKPSEGLSNGTSTSLFINLKPQSKPTFGSAFTNRPLLGTAEKSTNGEKPLSSSGAALSKPGNLEYNKQLTSLNCSVRDWIVKHVNANPLCDLTPIFKDYEKHLSAIEQKYGASSESGSESDGAAQTKTIPNLSSGKTVSIATFSFGNKDKAPEAPTKTPPDSKPQAAPTFNFGQKVDSSTLGLISSGGAPNFSFSIGAPSLFGKNNGSTASSSSSQESEPSGKTEEKGEGEEEEEPPKEVIQEIKEDDAFYSKKCKLFYKKDNEFKEKGVGTLHLKPVENKKTQLLVRADTNLGNILLNILVQPSMPCSRTGKNNVMIVCVPNPPVDEKNPTVPVTLLVRVKSAEDADQLHKILLEKKEV |
| Q9TXM1 | Q9TXM1_CAEEL | MSSSKPYPSGLPNSRRKRGGRRSSSRSNQESASNNMEHQITLDELFNPIAKQDSAQSTSREYGAKSGISHHGSVSFNGNTFMNGQQLNHSMTRHGRVFNQSMHAAQGNGSNAFNSIPPTAPVFSADFRRNLQTRNSSSWYERRFPVSTDQDDVQQSNTRRSRSRQNGQHGLSFSDGSNNYGHAGNKSFSVSSVPVGFQKQENNSKKLRQTNVHQQCLGNKSFNAQAGVHGHAFKKGHKDNKNASGKEVINSSLVQKHDAIKSRNLNQSFSGFPTHETSSMKNQQQKSRNDRKKSRGSSNFQDRTYFNTNDDELTDDVFIDDSMDAARGRRSRSVTKKLQQSTYSKQNAGSKQLTEKCKSSEEAAKRNLVSNVFSKDGTELSIEQLLEIVSMKIGQQIHLPSSSHGECSNLNRTLPASDLNCSIGEDFDSSFVDANNQTLPVSLPKKTSLSIKRRGSSRSASRLASLDVTLETVEEDEEPTPSPQPSSPPKISRRKWTGTFDANVEEMRRLLHGDPEMPKSANRASSSKDQINRNNVDVKRTPSSSIIPTPKALIGERCLTSSSKSSKLNKSLGVVDSKATKSPMYSVTVSGKETASGKRIAQKLTPKVVALESSYITGIPVSTDCNGCPTPKRSGINCEIRAAEVYNQAGKWPFEITSDPAPLPCESADRIEYPSQDCTQDPASTSPPPRISESLTAFLEAQQDFNDYIDTNYKEKTQLLKVNLNIHGMSPERWLYLNYFCTETIPRLDGPYADDPRVPPVRNMFRKWFLRFAEACLGNPHQLAVMQEIAATFVQARLDDTSSSTDSTNMLYMLWKECIGQKNIIAIADACLLAHLRKSDPIKYLNVKRDWLESIFDPPRDQ |
| P39936 | IF4F2_YEAST | MTDQRGPPPPHPQQANGYKKFPPHDNQYSGANNSQPNNHYNENLYSAREPHNNKQYQSKNGKYGTNKYNNRNNSQGNAQYYNNRFNNGYRLNNNDYNPAMLPGMQWPANYYAPQMYYIPQQMVPVASPPYTHQPLNTNPEPPSTPKTTKIEITTKTGERLNLKKFHEEKKASKGEEKNDGVEQKSKSGTPFEKEATPVLPANEAVKDTLTETSNEKSTSEAENTKRLFLEQVRLRKAAMERKKNGLISETEKKQETSNHDNTDTTKPNSVIESEPIKEAPKPTGEANEVVIDGKSGASVKTPQHVTGSVTKSVTFNEPENESSSQDVDELVKDDDTTEISDTTGGKTVNKSDDETINSVITTEENTVKETEPSTSDIEMPTVSQLLETLGKAQPISDIYEFAYPENVERPDIKYKKPSVKYTYGPTFLLQFKDKLKFRPDPAWVEAVSSKIVIPPHIARNKPKDSGRFGGDFRSPSMRGMDHTSSSRVSSKRRSKRMGDDRRSNRGYTSRKDREKAAEKAEEQAPKEEIAPLVPSANRWIPKSRVKKTEKKLAPDGKTELFDKEEVERKMKSLLNKLTLEMFDSISSEILDIANQSKWEDDGETLKIVIEQIFHKACDEPHWSSMYAQLCGKVVKDLDPNIKDKENEGKNGPKLVLHYLVARCHEEFEKGWADKLPAGEDGNPLEPEMMSDEYYIAAAAKRRGLGLVRFIGYLYCLNLLTGKMMFECFRRLMKDLNNDPSEETLESVIELLNTVGEQFEHDKFVTPQATLEGSVLLDNLFMLLQHIIDGGTISNRIKFKLIDVKELREIKHWNSAKKDAGPKTIQQIHQEEEQLRQKKNSQRSNSRFNNHNQSNSNRYSSNRRNMQNTQRDSFASTKTGSFRNNQRNARKVEEVSQAPRANMFDALMNNDGDSD |

*^a^* This protein set was curated by Vernon *et al.* ^1^.

**Table S2. Summary table for the set of proteins that exhibit homotypic phase separation behavior.**

| Seq. # | Seq. length | Σ class. dist.  P | longest PS  IDR | first residue longest PS  IDR | last residue longest PS  IDR | Σ class. dist. D | longest non-PS IDR | first residue longest non-PS IDR | last residue longest non-PS IDR | Σ class. dist.  F | longest F-labeled region | first residue longest F-labeled region | last residue longest F-labeled region | UniProtKB | Gene | Protein |
| --- | --- | --- | --- | --- | --- | --- | --- | --- | --- | --- | --- | --- | --- | --- | --- | --- |
| 1 | 592 | 1768 | 266 | 326 | 591 | 16 | 0 | 0 | 0 | 38 | 27 | 242 | 268 | Q92804 | RBP56 | TATA-b |
| 2 | 320 | 514 | 129 | 191 | 319 | 40 | 23 | 74 | 96 | 97 | 74 | 107 | 180 | P09651-2 | ROA1 | Isoform A |
| 3 | 708 | 952 | 214 | 1 | 214 | 12 | 0 | 0 | 0 | 432 | 332 | 319 | 650 | D0PV95 | DDX3 | ATP-depe |
| 4 | 263 | 1067 | 262 | 1 | 262 | 0 | 0 | 0 | 0 | 0 | 0 | 0 | 0 | H3BNZ4 | H3BNZ4 | FUS RNA |
| 5 | 662 | 461 | 95 | 47 | 141 | 37 | 0 | 0 | 0 | 424 | 329 | 259 | 587 | O00571 | DDX3X | ATP-depe |
| 6 | 724 | 459 | 234 | 23 | 256 | 31 | 0 | 0 | 0 | 537 | 452 | 262 | 713 | Q9NQI0 | DDX4 | Probable |
| 7 | 248 | 205 | 77 | 122 | 198 | 46 | 27 | 194 | 220 | 82 | 74 | 46 | 119 | Q15056 | IF4H | Eukaryoti |
| 8 | 823 | 757 | 309 | 1 | 309 | 148 | 40 | 517 | 556 | 101 | 99 | 628 | 726 | P14907 | NSP1 | Nucleopor |
| 9 | 292 | 614 | 291 | 1 | 291 | 0 | 0 | 0 | 0 | 0 | 0 | 0 | 0 | F8WC90 | F8WC90 | EWS RN |
| 10 | 386 | 102 | 42 | 344 | 385 | 23 | 0 | 0 | 0 | 231 | 151 | 205 | 355 | P31483 | TIA1 | Cytotoxic |
| 11 | 786 | 603 | 50 | 104 | 153 | 202 | 25 | 364 | 388 | 118 | 34 | 1 | 34 | P15502 | ELN | Elastin |
| 12 | 341 | 762 | 157 | 184 | 340 | 42 | 20 | 76 | 95 | 93 | 75 | 1 | 75 | P22626-2 | ROA2 | Isoform A |
| 13 | 323 | 231 | 94 | 1 | 94 | 41 | 0 | 0 | 0 | 212 | 189 | 134 | 322 | P22232 | FBRL | rRNA 2'- |
| 14 | 693 | 330 | 82 | 611 | 692 | 101 | 29 | 549 | 577 | 596 | 465 | 1 | 465 | G5EBV6 | PGL3 | Guanyl-sp |
| 15 | 172 | 235 | 98 | 74 | 171 | 4 | 0 | 0 | 0 | 33 | 64 | 1 | 64 | Q14011 | CIRBP | Cold-indu |
| 16 | 1105 | 2224 | 361 | 1 | 361 | 118 | 0 | 0 | 0 | 303 | 141 | 964 | 1104 | D3KYQ3 | D3KYQ3 | Macronuc |
| 17 | 1553 | 1540 | 640 | 1 | 640 | 122 | 0 | 0 | 0 | 898 | 541 | 1012 | 1552 | Q387F2 | Q387F2 | Nucleopor |
| 18 | 2834 | 4397 | 94 | 2646 | 2739 | 0 | 0 | 0 | 0 | 1029 | 117 | 2531 | 2647 | Q64K55 | Q64K55 | Aciniform |
| 19 | 959 | 2000 | 701 | 1 | 701 | 57 | 43 | 747 | 789 | 232 | 161 | 798 | 958 | Q02629 | NU100 | Nucleopor |
| 20 | 1113 | 2004 | 632 | 164 | 795 | 216 | 41 | 920 | 960 | 241 | 130 | 958 | 1087 | Q02630 | NU116 | Nucleopor |
| 21 | 997 | 1706 | 626 | 1 | 626 | 93 | 0 | 0 | 0 | 164 | 107 | 841 | 947 | F4ID16 | NU98B | Nuclear p |
| 22 | 2053 | 2753 | 504 | 276 | 779 | 170 | 35 | 1171 | 1205 | 1119 | 599 | 1454 | 2052 | Q54EQ8 | NUP98 | Nuclear p |
| 23 | 414 | 402 | 79 | 335 | 413 | 58 | 22 | 1 | 22 | 251 | 96 | 96 | 191 | Q13148 | TADBP | TAR DN |
| 24 | 2037 | 1528 | 269 | 1768 | 2036 | 410 | 53 | 1164 | 1216 | 758 | 407 | 15 | 421 | Q9PVZ2 | Q9PVZ2 | Nucleopor |
| 25 | 1678 | 1399 | 320 | 189 | 508 | 209 | 25 | 1018 | 1042 | 883 | 227 | 1451 | 1677 | G5EEH9 | NUP98 | Nuclear p |
| 26 | 1817 | 1299 | 193 | 1 | 193 | 399 | 61 | 886 | 946 | 1186 | 649 | 1168 | 1816 | P52948 | NUP98 | Nuclear p |
| 27 | 1475 | 1353 | 291 | 1184 | 1474 | 219 | 32 | 1150 | 1181 | 290 | 62 | 716 | 777 | P49790 | NU153 | Nuclear p |
| 28 | 599 | 584 | 97 | 18 | 114 | 66 | 21 | 471 | 491 | 224 | 104 | 375 | 478 | Q5EAX5 | Q5EAX5 | MGC8499 |
| 29 | 464 | 137 | 60 | 404 | 463 | 191 | 57 | 224 | 280 | 99 | 94 | 1 | 94 | Q9JMD0-3 | ZN207 | Isoform 3 |
| 30 | 1834 | 1305 | 243 | 213 | 455 | 532 | 104 | 872 | 975 | 932 | 330 | 1504 | 1833 | C3XWA2 | C3XWA2 | Nuclear p |
| 31 | 1790 | 1214 | 290 | 214 | 503 | 538 | 94 | 873 | 966 | 1090 | 578 | 1212 | 1789 | J7I6Y1 | J7I6Y1 | Nuclear p |
| 32 | 1960 | 1137 | 254 | 344 | 597 | 302 | 46 | 1034 | 1079 | 1227 | 715 | 1245 | 1959 | Q9VCH5 | NUP98 | Nuclear p |
| 33 | 1547 | 1395 | 383 | 1164 | 1546 | 297 | 29 | 1017 | 1045 | 234 | 46 | 766 | 811 | K9ZRR1 | K9ZRR1 | Nup153 |
| 34 | 669 | 464 | 155 | 281 | 435 | 106 | 26 | 138 | 163 | 152 | 145 | 1 | 145 | Q96PK6 | RBM14 | RNA-bind |
| 35 | 535 | 378 | 119 | 14 | 132 | 9 | 0 | 0 | 0 | 313 | 382 | 153 | 534 | K9ZTJ6 | K9ZTJ6 | Nup54 |
| 36 | 452 | 164 | 57 | 395 | 451 | 116 | 44 | 92 | 135 | 114 | 96 | 1 | 96 | Q7ZXV8 | ZN207 | BUB3-int |
| 37 | 547 | 513 | 167 | 1 | 167 | 59 | 0 | 0 | 0 | 150 | 113 | 349 | 461 | Q91349 | Q91349 | IL4I1 prot |
| 38 | 1050 | 744 | 65 | 571 | 635 | 181 | 42 | 431 | 472 | 327 | 101 | 1 | 101 | Q5EWX9 | Q5EWX9 | Nuclear p |
| 39 | 661 | 505 | 69 | 1 | 69 | 26 | 0 | 0 | 0 | 213 | 69 | 143 | 211 | P34761 | WHI3 | Protein W |
| 40 | 491 | 429 | 63 | 87 | 149 | 15 | 0 | 0 | 0 | 114 | 90 | 154 | 243 | Q5XGN1 | NUP42 | Nucleopor |
| 41 | 440 | 169 | 32 | 113 | 144 | 272 | 35 | 1 | 35 | 171 | 61 | 354 | 414 | Q6DEC7 | Q6DEC7 | LOC5036 |
| 42 | 862 | 269 | 62 | 140 | 201 | 256 | 46 | 328 | 373 | 317 | 166 | 696 | 861 | Q9TXM1 | Q9TXM1 | Uncharact |
| 43 | 914 | 188 | 79 | 17 | 95 | 572 | 87 | 147 | 233 | 413 | 132 | 694 | 825 | P39936 | IF4F2 | Eukaryoti |

**Supporting Figures**


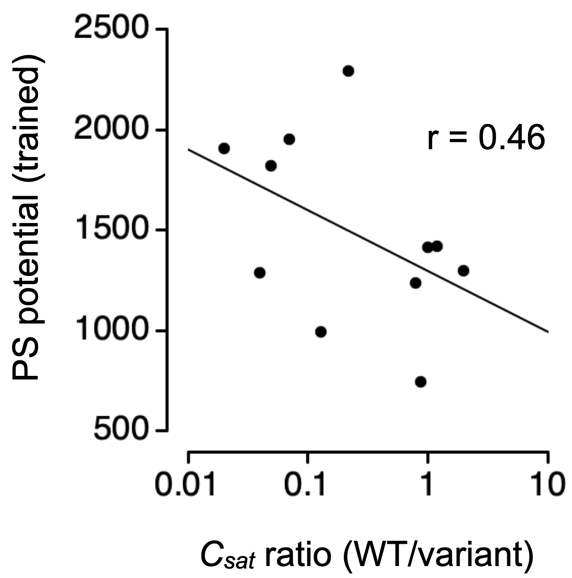


**Figure S1. Mutation effects on experimental *c_sat_* compared to the expanded PS potential trained previously using experimental ∆*h°* from a mutant dataset.** Experimental *c_sat_* ratio (x-axis) was digitally extracted from Figure S18A in Rekhi *et al.* ^2^; the sequence-calculated PS potential includes the summed P classifier distance plus *U_π_* and *U_q_* trained previously ^3^.

**Supporting References**

1. Vernon RM, Chong PA, Tsang B, Kim TH, Bah A, Farber P, Lin H, Forman-Kay JD (2018) Pi-Pi contacts are an overlooked protein feature relevant to phase separation. Elife 7.

2. Rekhi S, Garcia CG, Barai M, Rizuan A, Schuster BS, Kiick KL, Mittal J (2023) Expanding the molecular language of protein liquid-liquid phase separation. :2023.03.02.530853. Available from: https://www.biorxiv.org/content/10.1101/2023.03.02.530853v1

3. Ibrahim AY, Khaodeuanepheng NP, Amarasekara DL, Correia JJ, Lewis KA, Fitzkee NC, Hough LE, Whitten ST (2023) Intrinsically disordered regions that drive phase separation form a robustly distinct protein class. J Biol Chem 299:102801.
